# Antiproliferative effects of two gold(I)-N-heterocyclic carbene complexes in A2780 human ovarian cancer cells: a comparative proteomic study

**DOI:** 10.18632/oncotarget.25556

**Published:** 2018-06-15

**Authors:** Francesca Magherini, Tania Fiaschi, Elisa Valocchia, Matteo Becatti, Alessandro Pratesi, Tiziano Marzo, Lara Massai, Chiara Gabbiani, Ida Landini, Stefania Nobili, Enrico Mini, Luigi Messori, Alessandra Modesti, Tania Gamberi

**Affiliations:** ^1^ Department of Experimental and Clinical Biomedical Sciences “Mario Serio”, University of Florence, Florence, Italy; ^2^ Department of Chemistry “Ugo Schiff”, University of Florence, Florence, Italy; ^3^ Department of Chemistry and Industrial Chemistry, University of Pisa, Pisa, Italy; ^4^ Department of Experimental and Clinical Medicine, University of Florence, Florence, Italy; ^5^ Department of Health Sciences, University of Florence, Florence, Italy

**Keywords:** gold(I)-N-heterocyclic carbene complexes, A2780 human ovarian cancer cells, proteomics, mitochondrial metabolism, thioredoxin reductase

## Abstract

Au(NHC) and Au(NHC)_2_, *i.e.* a monocarbene gold(I) complex and the corresponding bis(carbene) complex, are two structurally related compounds, endowed with cytotoxic properties against several cancer cell lines. Herein, we explore the molecular and cellular mechanisms at the basis of their cytotoxicity in A2780 human ovarian cancer cells. Through a comparative proteomic analysis, we demonstrated that the number of modulated proteins is far larger in Au(NHC)_2_-treated than in Au(NHC)-treated A2780 cells. Both gold compounds mainly affected proteins belonging to the following functional classes: protein synthesis, metabolism, cytoskeleton and stress response and chaperones. Particularly, Au(NHC)_2_ gave rise to an evident upregulation of several glycolytic enzymes. Moreover, only Au(NHC)_2_ triggered a net impairment of respiration and a metabolic shift towards glycolysis, suggesting that mitochondria are relevant cellular targets. We also found that both carbenes, similarly to the gold(I) compound auranofin, caused a strong inhibition of the seleno-enzyme thioredoxin reductase (TrxR). In conclusion, we highlighted that coordination of two carbene ligands to the same gold(I) center greatly enhances the antiproliferative effects of the resulting compound in comparison to the monocarbene derivative. Moreover, TrxR inhibition and metabolic impairment seem to play a major role in the Au(NHC)_2_ cytotoxicity. Overall, these antiproliferative effects were also confirmed on other two human ovarian cancer cell lines (*i.e.* SKOV3 and IGROV1).

## INTRODUCTION

Ovarian cancer is the 7^th^ most common cancer in women worldwide with an estimated incidence rate of 6.1 per 100,000 [1]. In addition, it represents the 8t^h^ cause of death from cancer in women worldwide [1]. Such dismal prognosis is due to several factors, including that most of ovarian cancer cases are diagnosed with advanced disease [2]. Ovarian cancer chemotherapy is mainly represented by combinations of carboplatin and paclitaxel both in the adjuvant and metastatic settings. Although ovarian cancer is considered a chemosensitive cancer, tumor drug resistance develops in most of cases. To date, in addition to other cytotoxic drugs, several targeted agents such as PARP inhibitors (e.g. olaparib, rucaparib) or the anti VEGF MoAb bevacizumab may be used in ovarian cancer patients whose disease recurred [3]. However, despite the availability of such new treatment options, five-year overall survival of all stage ovarian cancer is below 50% [1, 4].

Thus, there is a strong need for novel, highly effective therapies for the treatment of advanced epithelial ovarian cancer. The discovery of cisplatin's antitumor activity in 1969 prompted the search for novel metal-containing complexes as potential anticancer drugs. Among the several non-platinum drugs considered so far, gold-based compounds are gaining ever-growing attention. Gold has been largely used in medicine since very ancient times. Currently, the gold(I) complex auranofin, (1-thio-β-D-glucopyranose-2,3,4,6-tetraacetato-S) (triethylphosphine) gold(I)), is clinically used in severe forms of rheumatoid arthritis, and its anticancer potential was already described in both *in vitro* and in *vivo* models [5, 6]. Moreover, clinical trials including auranofin are currently ongoing also in ovarian cancer patients [7, 8].

Overall, gold compounds constitute a variegate family of very promising experimental agents for cancer treatment. Indeed, several gold(I) and gold(III) complexes were recently shown to manifest outstanding antiproliferative properties *in vitro* against selected human cancer cell lines, and some of them performed remarkably well even in *in vivo* cancer models [9, 10]. As previously mentioned, investigations on the cytotoxicity scores of gold complexes were initially focused on auranofin and its analogues, which present linear gold phosphane structures [11, 12]. More recently, a variety of gold derivatives has been tested as potential antitumor agents, including organogold derivatives, complexes with polydentate nitrogen donor ligands, gold porphyrins, gold dithiocarbamates, and gold-N-heterocyclic carbene (NHC) [13–17]. Based on the great structural variety of the used ligands and their role in controlling the reactivity of the gold centre, a unique mode of action or pharmacological profile is unlikely to exist. Gold compounds can trigger cell death through a multitude of mechanisms by affecting mitochondria and the redox balance, by modulating cell cycle, by controlling proteolysis and signal transduction [18–23]. Though the detailed mechanisms of action remain unclear, the inhibition of the seleno-enzyme thioredoxin reductase (TrxR) seems to be a common mechanistic trait to explain, at least partially, the cytotoxic actions of several gold(I) and gold(III) complexes, as strong TrxR inhibition may eventually lead to cancer cell apoptosis through activation of a mitochondrial pathway [24–28].

N-Heterocyclic carbenes (NHCs) are very interesting gold(I) ligands as they manifest donor properties similar to phosphines, thus affording a very stable gold(I) coordination. Hydrophilic/lipophilic properties can be readily fine-tuned by the incorporation of appropriate functional groups on the carbene moieties. Within this frame, several gold carbene complexes were prepared and characterized during the past few years that turned out particularly effective and promising from the biological and pharmacological point of view [29–34]. Even though several studies have been carried out so far on the cellular effects of gold carbene compounds and valuable mechanistic information has been gathered, the precise mode of action of gold carbene complexes, at the molecular level, is still largely unclear. Based on the observations reported so far, gold carbene complexes are mainly considered as a class of anti-mitochondrial agents [35]. Indeed, recent studies have demonstrated a strong selective TrxR inhibition by several gold(I)–NHC complexes [26, 35–39]. Similarly, Holenya et *al*. [40] outlined thioredoxin reductase as a central target for a new gold(I) carbene complex. Its inhibition may trigger a severe metabolic impairment accompanied by activation of pro-apoptotic signalling, resulting in cell death.

**Figure 1:**
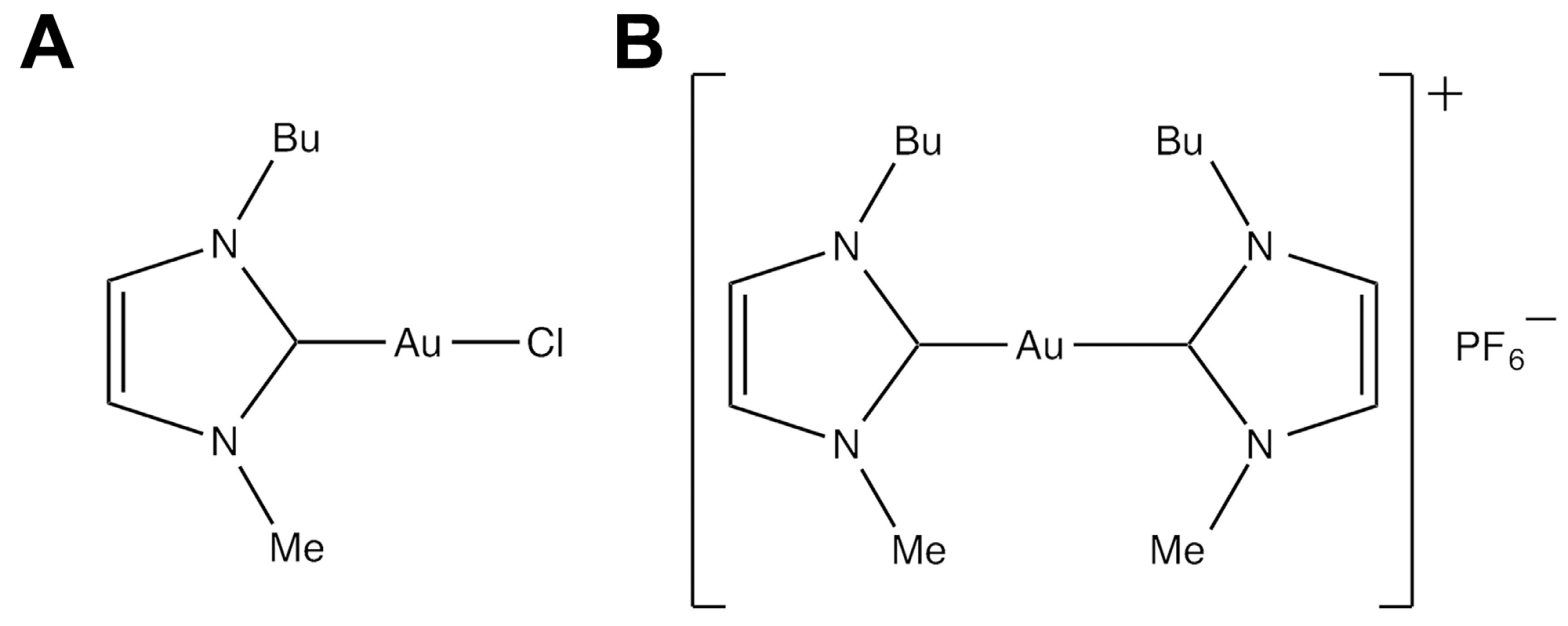
Chemical structure of gold(I)-N-heterocyclic carbene complexes **(A)** Au(NHC) and **(B)** Au(NHC)_2_.

Within this frame, the present work explored the cytotoxic activity of two new gold(I) carbene complexes, previously characterized from the chemical point of view (Figure 1) [41]. These two structurally related compounds are characterised by the presence of one (complex **1**: Au(NHC)Cl, *i.e.* Au(NHC)) or two (complex **2**:[Au(NHC)_2_]PF_6_, *i.e.* Au(NHC)_2_) 1-butyl-3-methyl-imidazole-2-ylidene moieties acting as NHC ligand coordinating the gold(I) centre, with **1** bearing a chloride as the second ligand in place of the second NHC. This difference renders the two compounds highly distinct even in terms of the overall charge as compound **2** is mono-cationic while compound **1** is neutral. In complex **1** the second gold(I) ligand is a chloride ion that, in principle, is believed to act as the labile ligand.

Herein, the biological behaviour of these two gold carbene complexes has been analysed in A2780 human ovarian cancer cell line, according to the following strategy. First, an extensive proteomic investigation study has been carried out according to classical procedures aimed at highlighting differences in protein expression. Then, these differences were analysed through bioinformatics tools leading to the identification of specific components of the cell machinery that appear to be perturbed by gold treatment. Finally, the suggested molecular and cellular effects were confirmed through direct measurements of cell metabolic activities and cell functioning. Relevant differences were highlighted in the cellular effects produced by the two-investigated gold(I) carbene compounds that might be associated to significant mechanistic differences in the mode of action. These results were also confirmed on other two human ovarian cancer cell lines (*i.e.* SKOV3 and IGROV1).

## RESULTS

### Lipophilicity, cytotoxicity, cell cycle alterations and cell death

Lipophilicity expressed by the octanol-water partition coefficient as the logarithm of the concentration ratio (logP) of a compound in each phase constituting the binary immiscible mixture, represents one of the most important molecular features for the drug action profile, influencing both pharmacokinetic and pharmacodynamics processes. LogP value of the two gold(I) carbene complexes, Au(NHC)Cl and [Au(NHC)_2_]PF_6_, has been measured by modification of the reported shake-flask method (Table 1) [42]. The reported values show that the neutral complex Au(NHC)Cl (*i.e.* Au(NHC)) has a far greater lipophilicity than the cationic complex [Au(NHC)_2_]PF_6_(*i.e.* Au(NHC)_2_) in accord with expectations, since the positive charge of bis(carbene) species greatly increases its affinity for water.

**Table 1:** Measured logP values for carbene 1 and 2

	Compound	logP
**1.**	Au(NHC)Cl	2.12
**2.**	[Au(NHC)_2_]PF_6_	0.06

**Figure 2:**
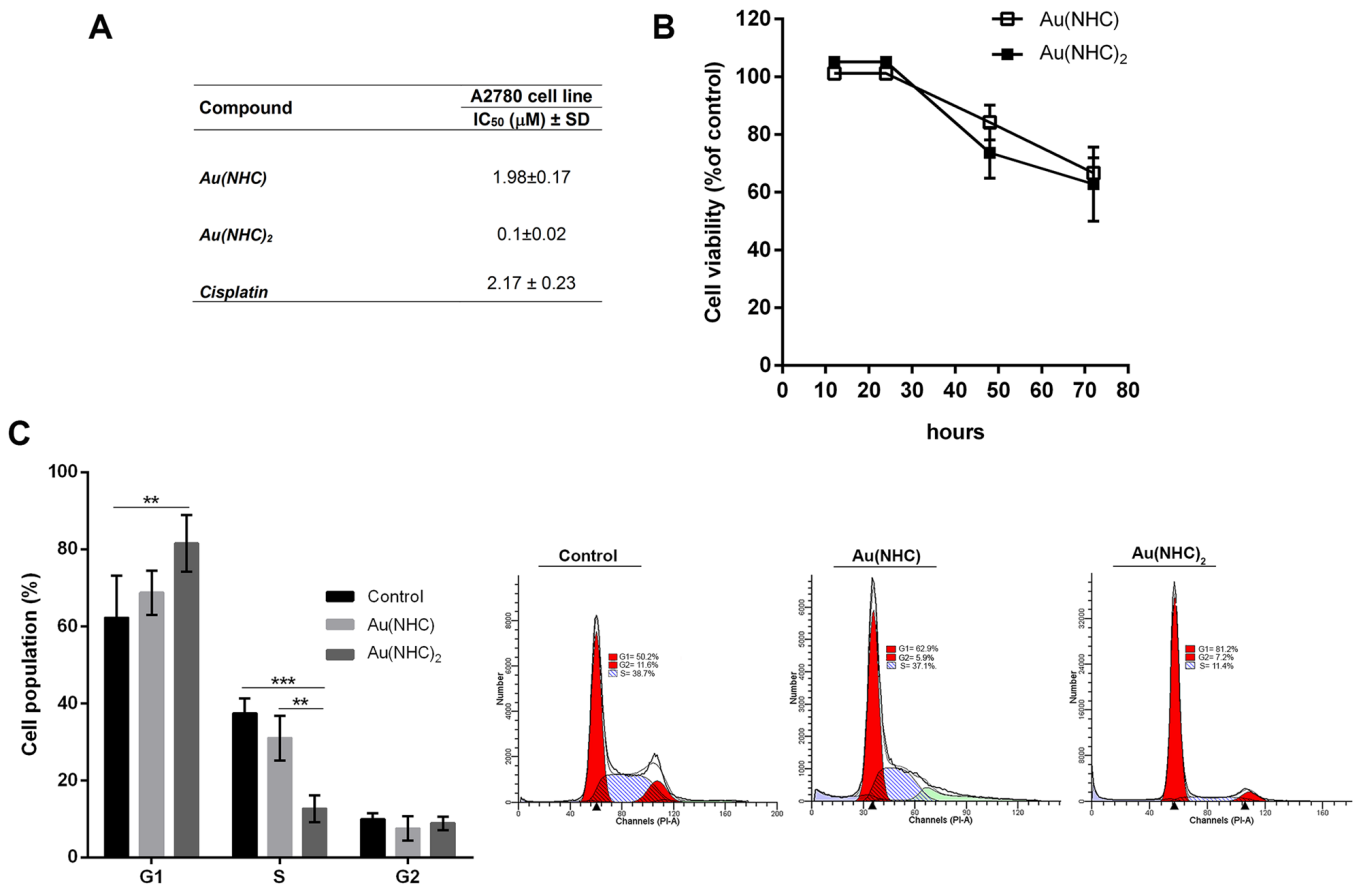
Antiproliferative activity of Au(NHC) and Au(NHC)_2_ against A2780 human ovarian cancer cells **(A)** Inhibitory concentration IC_50_ (μM) values were obtained after 72 h of treatment according to the procedure described by Skehan et *al.* [43]. The IC_50_ value of cisplatin was also assessed. **(B)** Cell viability time course upon Au(NHC) and Au(NHC)_2_ treatment using MTT assay. Values were obtained by measuring the percentage of treated-A2780 viable cells relative to untreated controls after 12, 24, 28, 72 h of incubation with the 72 h-exposure IC_50_-dose. **(C)** Cell cycle distribution was analysed by flow cytometric analysis. Histograms reported the cell percentage in G1, S and G2 phase of cell cycle after 48 h of treatment with the 72 h-exposure IC_50_-dose of Au(NHC) and Au(NHC)_2_. Flow cytometric images are representative of three independent experiments. All the experiments were performed at least in triplicate and results are reported as mean ± SD. The statistical analysis was carried out using one-way ANOVA test followed by Tuckey's multiple comparisons test using Graphpad Prism v 6.0 (^**^p<0.01, ^***^p<0.001).

The cytotoxic activity of these gold(I) complexes was evaluated in A2780 human ovarian carcinoma cell line by cell proliferation assay according to the procedure described by Skehan et *al.* [43] and compared with that of cisplatin. The IC_50_ values, observed after 72 h exposure to each carbene complex and cisplatin, are reported in Figure 2A. The monocarbene complex Au(NHC) showed an inhibitory potential similar to that of cisplatin, giving rise to IC_50_ values in the micromolar range (ca. 2 μM). Remarkably, the bis(carbene) complex Au(NHC)_2_ displayed a far more pronounced cytotoxicity with IC_50_ value in the high nanomolar range (ca. 0.1 μM). Besides, the antiproliferative effect of the carbene complexes in A2780 was evaluated by a cell viability assay using 3-(4,5-dimethyl-thiazol-2-yl)-2,5-diphenyltetrazolium bromide (MTT) that is reduced to violet formazan in viable cells. For each complex, a time course at 12, 24, 48 and 72 h drug exposure with a concentration corresponding to their 72 h-IC_50_-dose, was carried out (Figure 2B). The obtained results were in line with those of IC_50_ experiments. Indeed, the bis(carbene) Au(NHC)_2_ achieved the decrease of 50% cell viability, after 72 h of incubation, with a dose about 20-fold lower than the monocarbene complex Au(NHC).

**Figure 3:**
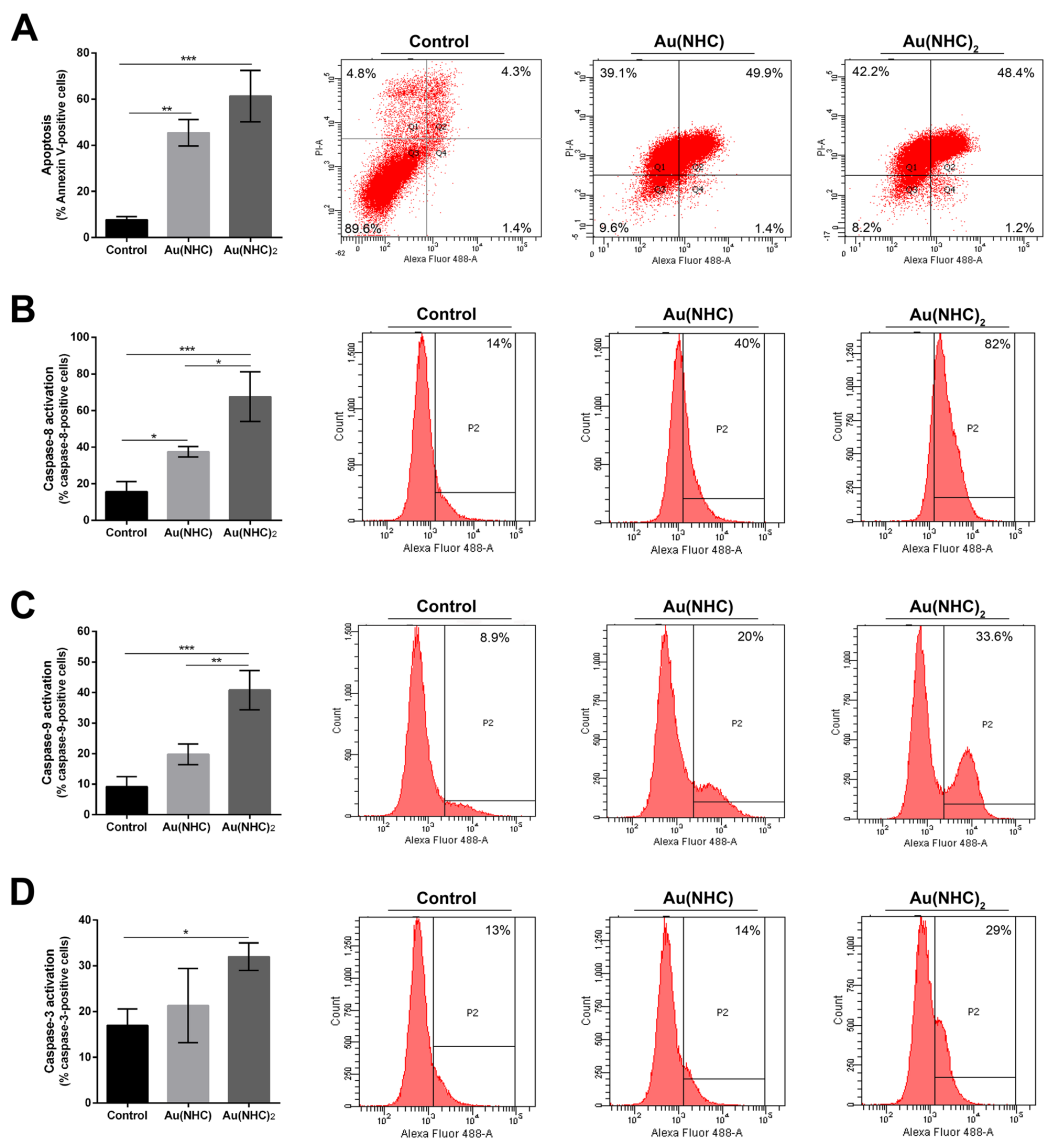
Apoptosis and modulation of cell signalling in A2780 cell line **(A)** Percentage of apoptotic cells shown by flow cytometry analysis of annexin V/propidium iodide-stained A2780 cells treated for 72 h with Au(NHC) and Au(NHC)_2_ -72 h-exposure IC_50_-dose. **(B)** Caspase-8, **(C)** Caspase-9 and **(D)** Caspase-3 activity shown by fluorescence-activated cell sorting analysis using FAM FLICA in A2780 cells treated for 72 h with Au(NHC) and Au(NHC)_2_ concentration corresponding to their 72 h-exposure IC_50_-dose. Flow cytometric images are representative of three independent experiments. Histograms report the mean values ±SD. The statistical analysis was carried out using one-way ANOVA test followed by Tuckey's multiple comparisons test using Graphpad Prism v 6.0 (^*^p<0.05, ^**^p<0.01, ^***^p<0.001).

Afterwards, the effects of carbene complexes on the A2780 cell cycle progression were analysed by propidium iodide staining and flow cytometry, after 24, 48 and 72 h of treatment. In the first 24 h, there were no significant differences between carbene-treated and untreated cells (data not shown). After 48 h, Au(NHC)_2_ induced a significant accumulation of cells in the G_1_ phase (about 80%) followed by a decrease of cell population in S phase (about 11%) suggesting an associated cell cycle arrest (Figure 2C). Conversely, the behaviour of Au(NHC)-treated cells was similar to that of control cells with about 50% of cells in G_1_ phase and 38% in S phase. After 72 h, the cell cycle analysis highlighted that Au(NHC)_2_ and, with smaller extent, Au(NHC) were able to induce DNA fragmentation corresponding to the appearance of sub-G0/G1 population (Supplementary Figure 1). This result suggested that A2780 cancer cells could undergo apoptosis under carbene treatment as reported for cisplatin and the gold(I) auranofin [25]. Therefore, we examined if the selected mono and bis(carbene) complexes also favour apoptosis as the main way for cell death. We performed flow cytometry analysis of annexin V/propidium iodide-stained A2780 cells treated with Au(NHC) or Au(NHC)_2_ for 72 h. After treatment, the cell-state distribution revealed a pronounced antiproliferative effect. As shown in Figure 3A, about 50-60% of late apoptotic cells were present in both carbene-treated cells in comparison to control cells. The apoptosis induction by Au(NHC) and Au(NHC)_2_ was supported by western blot analysis of the pro-apoptotic Bax and the anti-apoptotic Bcl2 protein amount. In accord with the flow cytometry analysis of apoptosis, after 72 h of treatment, both Au(NHC) and Au(NHC)_2_ triggered an increase of Bax protein level along with a decrease of Bcl2 protein level (Supplementary Figure 2). To elucidate whether apoptosis was induced by extrinsic or intrinsic apoptotic signalling pathway, we assessed the activation of specific caspases. After 72 h of treatment, we analysed by flow cytometry the activation of the initiator caspase-8 (extrinsic pathway) and caspase-9 (intrinsic pathway) along with the activation of the effector caspase-3. As shown in Figure 3B, 3C and 3D, both carbene complexes, with a stronger effect for bis(carbene), activated the initiator caspase-8. Only Au(NHC)_2_ triggered a significant increased activity of the initiator caspase-9 and the effector caspase-3.

### Proteomic profile of Au(NHC) and Au(NHC)_2_ -treated A2780 cells

**Figure 4:**
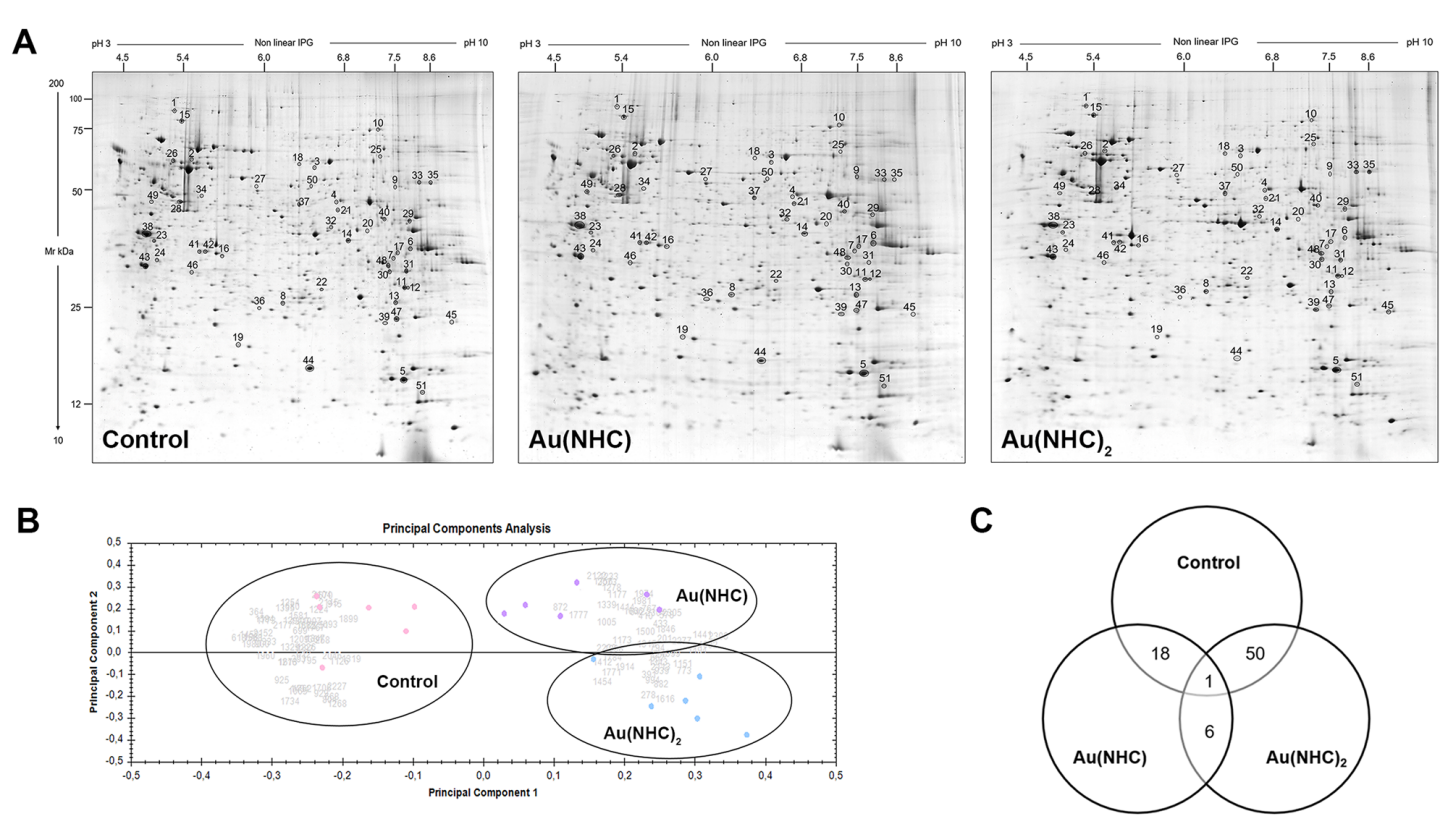
Proteomic profile of Au(NHC) and Au(NHC)_2_-treated A2780 cells **(A)** Representative colloidal Coomassie blue silver-stained 2-DE gel images for control, Au(NHC) and Au(NHC)_2_-treated A2780 cells. Proteins (700 μg) were separated by 2-DE using IPG strips with a pH gradient of 3–10 non-linear and 9–16% linear gradient SDS-PAGE, as described in detail in Materials and Methods. Black circles and numbers indicate statistically differentially abundant protein spots, given as in Table 2. **(B)** Multivariate analysis of the 2-DE gel images results using Principal Components Analysis (PCA) performed by Progenesis SameSpots 4.0 software (Nonlinear Dynamic, UK). **(C)** Distribution of the differentially abundant protein spots between pairwise comparisons of control cells, Au(NHC)-treated cells and Au(NHC)_2_-treated cells, as detected by 2-DE analysis.

To further characterize the cytotoxic and antiproliferative mechanism of the mono and bis(carbene) complex, proteome profiles of control, Au(NHC) and Au(NHC)_2_ -treated cells were studied by comparative 2-DE based proteomic analysis. A2780 cells were treated for 24 h with mono or bis(carbene) at a concentration corresponding to their 72 h-exposure IC_50_-dose (2 μM and 0.1 μM, respectively). At this time, cells are viable (as shown in MTT time course experiment) and proteomic study can allow the early effects of treatments to emerge. After carbene treatment, cellular protein extracts were prepared and separated by 2-D gel electrophoresis as reported in Materials and Methods. In Figure 4A representative 2-DE gels of control, Au(NHC) and Au(NHC)_2_ -treated A2780 cells are shown. The 2-DE gel images were analysed by Progenesis SameSpots software 4.0 (Nonlinear Dynamics, UK) using default parameters. After automatic spot detection, an average of about 1,500 protein spots was detected in each gel. The computational 2-DE gel image analysis pointed out 70 differentially expressed protein spots (ANOVA *p-*value≤0.05). In addition to univariate analysis (ANOVA test), multivariate analyses (*q*-value, PCA and power analysis) were performed to explore categories of differential protein expression. In detail, to reduce the expected level of false positives we performed a statistical analysis on the ANOVA *p-*values, by applying the false discovery rate correction method known as *q*-value, using Progenesis SameSpots software 4.0 [44]. To obtain an overview of the proteomic data for overall trends in carbene-treated and control cells a multivariate analysis by PCA was performed. Gels were grouped according to the variance of their protein spot expression. As shown in Figure 4B the first principal component, which distinguished 39% of the variance, clearly separates the proteome data of the mono and bis(carbene)-treated cells from control cells, and the second component, with additional 14% of the variance, distinguished between the two gold NHC complexes. Moreover, we calculated the power of our statistical analysis [45]. In our experiments, we achieved a target power of 87% confirming that the number of sample replicates used was appropriate. Finally, we considered as statistically different expressed the protein spots with an adjusted *q*-value≤0.1 and a power ≥0.8. Therefore, a statistically different expression level was found for 51 protein spots between the three cell lines. Afterwards, the ANOVA *p*-values have been submitted to Tukey post-hoc analysis for multiple comparison using GraphPad Prism 6.0 software. Comparisons between pairwise A2780 control cells, Au(NHC), and Au(NHC)_2_-treated cells, revealed that the number of modulated spots was higher between controls and Au(NHC)_2_-treated cells (51 protein spots) (Figure 4C). Among these 51 protein spots, 19 were also significantly different between monocarbene and control cells showing the same trend as bis(carbene); six spots differed between Au(NHC) and Au(NHC)_2_-treated cells and finally only one protein spot varied between all the three conditions (Figure 4C and Table 2). In Supplementary Figure 3A–3E histograms representing spot abundance variation between each treatment are reported. It is interestingly to note that, although not significant, the trend between control and monocarbene-treated cells was always similar to that observed for bis(carbene) with the exception of the six spots that displayed the same trend as the control and thus resulted significant different from bis(carbene) treated cells.

**Table 2:** Differentially expressed protein spots identified by MALDI TOF/TOF mass spectrometry analysis

Spot No^a^	Protein name	Gene Name	AC^b^	Observed Theorical		Mascot search results			A NOVA *p*-value^g^	*FDR*^h^	*Tukey's post-hoc test ^i^/ Average ratio^j^*		
				Mr (kDa)/ pI^c^	Mr (kDa)/ pI	PMF Score^d^	Matched Pept.^e^	Seq. coverage (%)^f^			Au(NHC) *vs* CTR	Au(NHC)_2_ *vs* CTR	Au(NHC)_2_ *vs* Au(NHC)
***Stress Response and Chaperones***													
**1**	Heat shock 70 kDa protein 4	HSPA4	P34932	94.2/5.1	95.1/5.1	62^k^	7/15	11	0.030	0.081	ns	(^*^) / 1.4	ns
**2**	Stress-70 protein, mitochondrial	HSPA9	P38646	63.0/5.2	73.9/5.9	62^k^	6/13	12	0.002	0.024	(^**^) / -1.6	(^*^) / -1.4	ns
**3**	T-complex protein 1 subunit zeta	CCT6A	P40227	58.0/6.4	57.9/6.2	155	27/93	48	0.020	0.083	ns	(^*^) / 1.5	ns
**4**	Stress-induced-phosphoprotein 1	STIP1	P31948	46.0/6.6	45.9/6.6	177	32/86	51	0.03	0.082	ns	(^*^) / -1.5	ns
**5**	Peptidyl-prolyl cis-trans isomerase A	PPIB	P62937	16.4/7.6	18.2/7.7	79	11/55	50	0.0007	0.029	(^*^) / -1.2	(^***^) / -1.3	ns
***Metabolism***													
***(Glucose Metabolism)***													
**6**	Glyceraldehyde-3-phosphate dehydrogenase	GAPDH	P04406	37.2/7.6	36.2/8.6	109	13/23	40	0.0081	0.044	ns	(^**^) / 1.5	ns
**7**	Fructose-bisphosphate aldolase A	ALDOA	P04075	35.1/7.5	39.8/8.3	69^k^	8/26	29	0.005	0.035	ns	(^*^) / 1.6	ns
**8**	Triosephosphate isomerase 1	TPI1	P60174	26.2/6.2	31.1/5.6	92	7/18	36	0.007	0.040	ns	(^*^) / 1.5	ns
**9**	UTP--glucose-1-phosphate uridylyltransferase	UGP2	Q16851	53.1/7.5	56.9/8.2	159	19p/64	25	0.034	0.083	ns	(^*^) / -1.5	ns
***(Cellular Respiration and ATP metabolism)***													
**10**	Aconitate hydratase, mitochondrial	ACO2	Q99798	81.6/7.2	86.1/7.4	90	10/30	22	0.0004	0.014	(^**^) / -1.4	(^***^) / -1.6	ns
**11**	Adenylate kinase 2, mitochondrial	AK2	P54819	28.9/7.5	26.7/7.7	62^k^	6/24	25	0.005	0.036	(^*^) / -1.3	(^**^) / -1.5	ns
**12**	Adenylate kinase 2, mitochondrial	AK2	P54819	28.9/7.6	26.7/7.7	62^k^	6/26	25	0.004	0.033	(^*^) / -1.4	(^**^) / -1.6	ns
**13**	ATP synthase subunit gamma, mitochondrial	ATP5C1	P36542	26.3/7.5	33.0/9.2	62^k^	6/28	15	0.003	0.081	(^*^) / -1.2	(^**^) / -1.4	ns
**14**	Cytochrome P450 4A22	CYP4A22	Q5TCH4	39.2/6.8	59.7/9.2	59^k^	7p/22	15	0.030	0.082	ns	(^*^) / -1.2	ns
**15**	Transitional endoplasmic reticulum ATPase	VCP	P55072	87.9/5.1	89.9/5.1	213	26/52	45	0.020	0.081	ns	(^*^) / -1.4	ns
**16**	Transitional endoplasmic reticulum ATPase (fragment)	VCP	P55072	35.6/5.5	89.9/5.1	74	9/38	15	0.030	0.085	ns	(^*^) / 1.8	ns
***(Folate Metabolism)***													
**17**	Bifunctional ethylenetetrahydrofolate dehydrogenase/cyclohydrolase, mitochondrial	MTHFD2	P13995	36.1/7.5	38.1/8.9	85	6/17	25	0.040	0.086	ns	(^*^) / -1.5	ns
***(Ketone Metabolism)***													
**18**	Succinyl-CoA:3-ketoacid coenzyme A transferase 1, mitochondrial	OXCT1	P55809	59.8/6.3	52.1/6.0	154	17/36	45	0.005	0.036	ns	(^**^) / 1.6	(^*^) / 1.5
***(Nucleotide Metabolism)***													
**19**	Deoxyuridine 5’-triphosphate nucleotidohydrolase, mitochondrial	DUT	P33316	20.1/5.7	26.8/9.5	72	6/21	34	0.00002	0.004	(^***^) / -1.7	(^***^) / -2.1	ns
***(Amino-acid Metabolism)***													
**20**	3-hydroxyisobutyryl-CoA hydrolase, mitochondrial	HIBCH	Q6NVY1	41.6/7.0	39.5/6.3	223	22/37	49	0.0024	0.036	ns	(^**^) / -1.4	(^*^) / -1.3
***Protein synthesis***													
**21**	Elongation factor Tu, mitochondrial	TUFM	P49411	44.7/6.6	49.9/7.3	73	6/12	17	0.0150	0.061	ns	(^*^) / 1.5	ns
**22**	Elongation factor 2 (fragment)	EEF2	P13639	28.5/6.5	95.2/6.4	107	22/67	25	0.001	0.021	ns	(^**^) / 1.8	(^*^) / 1.4
**23**	Elongation factor 1-delta	EEF1D	P29692	39.2/4.7	31.2/4.9	58^k^	6/32	30	0.0134	0.081	(^*^) / -1.3	(^*^) / -1.4	ns
**24**	WD repeat-containing protein 61	WDR61	Q9GZS3	34.6/4.8	33.7/5.2	75	6/19	24	0.04	0.086	ns	(^*^) / -1.4	ns
**25**	Heterogeneous nuclear ribonucleoprotein Q	SYNCRIP	O60506	64.2/7.2	69.5/8.7	200	22/45	40	0.02	0.081	ns	(^*^) / 1.8	ns
**26**	Heterogeneous nuclear ribonucleoprotein K	HNRNPK	P61978	62.0/5.1	51.2/5.4	121	13/29	35	< 0.0001	0.011	(^***^) / -1.4	(^****^) / -1.6	ns
**27**	Heterogeneous nuclear ribonucleoprotein K	HNRNPK	P61978	53.4/5.9	51.2/5.4	118	16/54	42	0.02	0.069	ns	(^*^) / 1.4	ns
**28**	Heterogeneous nuclear ribonucleoprotein F	HNRNPF	P52597	46.0/5.2	45.9/5.4	98	9/37	28	0.02	0.069	ns	(^*^) / -1.6	ns
**29**	Heterogeneous nuclear ribonucleoprotein D0	HNRNPD	Q14103	43.1/7.6	38.6/7.6	88	12/36	29	0.008	0.043	(^*^) / 1.6	(^*^) / 1.7	ns
**30**	Heterogeneous nuclear ribonucleoproteins A2/B1	HNRNPA2B1	P22626	32.2/7.4	37.5/8.9	66^k^	7/27	18	0.0009	0.021	(^*^) / 1.5	(^***^) / 1.8	ns
**31**	Heterogeneous nuclear ribonucleoprotein A1	HNRNPA1	P09651	32.2/7.6	38.8/9.2	142	17/58	32	0.0070	0.04	(^*^) / 2.2	(^*^) / 2.4	ns
**32**	RNA-binding protein 4	RBM4	Q9BWF3	42.2/6.6	40.7/6.6	108	9/22	36	0.0107	0.058	(^*^) / -1.3	(^*^) / -1.4	ns
**33**	Zinc finger protein 486	ZNF486	Q96H40	54.1/8.1	55.1/9.3	66^k^	8/28	22	0.019	0.067	ns	(^*^) / 2.2	ns
**34**	Zinc finger protein 18	ZNF18	P17022	48.6/5.3	62.3/5.7	66^k^	8p/57	20	0.0007	0.029	ns	(^***^) / 1.6	(^**^) / 1.5
**35**	Plasminogen activator inhibitor 1 RNA-binding protein	SERBP1	Q8NC51	54.1/8.6	45.0/8.7	66^k^	9/26	25	0.0207	0.069	ns	(^*^) / 2.1	ns
**36**	N-myc (and STAT) interactor	NMI	Q13287	25.4/5.9	35.2/5.2	77	8/21	26	0.044	0.089	ns	(^*^) / -1.4	ns
***Cytoskeleton and Cell Structure***													
**37**	Proliferation-associated protein 2G4	PA2G4	Q9UQ80	45.6/6.3	44.1/6.1	95	8/18	28	0.0159	0.060	ns	(^*^) / -1.5	(^*^) / -1.5
**38**	Nucleophosmin	NPM1	P06748	41.1/4.6	32.7/4.6	94	11/40	31	0.0002	0.012	(^*^) / -1.4	(^***^) / -2.5	(^*^) / -1.8
**39**	Mitogen-activated protein kinase kinase kinase MLT (fragment)	ZAK	Q9NYL2	23.1/7.3	91.2/7.9	60^k^	9/41	17	0.0370	0.086	ns	(^*^) / 1.4	ns
**40**	26S protease regulatory subunit 10B	PSMC6	P62333	43.4/7.3	44.4/7.1	198	23/35	58	0.0370	0.086	ns	(^*^) / -1.2	ns
**41**	Tubulin beta chain	TUBB	P07437	36.6/5.3	50.1/4.8	72	6/15	21	0.0040	0.033	(^*^) / -1.4	(^**^) / -1.6	ns
**42**	F-actin-capping protein subunit alpha-1	CAPZA1	P52907	36.4/5.3	33.1/5.4	96	6/8	34	0.035	0.082	ns	(^*^) / -1.2	ns
**43**	Tropomyosin alpha-3 chain	TPM3	P06753	33.2/4.6	32.9/4.7	70^k^	6/16	14	0.024	0.072	ns	(^*^) / -1.3	ns
**44**	Cofilin-1	COF1	P23528	17.4/6.4	18.7/8.2	122	11/43	68	0.0005	0.019	(^**^) / -3.1	(^***^) / -4.3	ns
**45**	Cytoskeleton-associated protein 2 (fragment)	CKAP2	Q8WWK9	23.2/9.6	77.5/9.5	81	14/32	18	0.0035	0.031	(^*^) / 1.5	(^**^) / 1.6	ns
**46**	Vascular cell adhesion protein 1 (fragment)	VCAM1	P19320	31.9/5.2	82.3/5.1	63^k^	6/15	16	0.034	0.036	ns	(^**^) / 1.7	(^*^) / 1.4
***Cell redox homeostasis***													
**47**	Peroxiredoxin-1	PRDX1	Q06830	23.7/7.5	22.3/8.3	79	7/40	52	0.016	0.049	(^*^) / -1.3	(^*^) / -1.4	ns
***Transport***													
**48**	Voltage-dependent anion-selective channel protein 2	VDAC2	P45880	33.3/7.3	32.1/7.5	75	6/20	35	0.0032	0.030	(^**^) / 1.4	(^**^) / 1.4	ns
**49**	Oxysterol-binding protein-related protein 1 (fragment)	OSBPL1A	Q9BXW6	46.1/4.7	109.8/5.9	57^k^	7/13	8	0.005	0.035	ns	(^**^) / 2	ns
***DNA replication and repair***													
**50**	RuvB-like 1	RUVBL1	Q9Y265	53.4/6.4	50.2/6.0	269	30/63	61	0.0220	0.091	ns	(^*^) / -1.4	ns
**51**	Single-stranded DNA-binding protein, mitochondrial	SSBP	Q04837	15.2/8.2	17.2/9.6	70^k^	6/23	37	0.0122	0.067	ns	(^**^) / 1.5	ns

^a^ Spot numbers match those reported in the representative 2-DE images shown in Figure 4A.

^b^ Accession number in Swiss-Prot/UniProtKB (http://www.uniprot.org/).

^c^ Observed Mr/pI, based on the calculation using Progenesis SameSpots 4.0 software

^d^ MASCOT MS score (Matrix Science, London, UK; http://www.matrixscience.com). MS matching score greater than 56 was required for a significant MS hit (*p*-value<0.05).

^e^ Number of matched peptides correspond to peptide masses matching the top hit from Ms-Fit PMF, searched peptides are also reported.

^f^ Sequence coverage = (number of the identified residues/total number of amino acid residues in the protein sequence) x100%.

^g^ ANOVA test was performed by Progenesis SameSpots 4.0 software to determine if the relative change was statistically significant (*p*<0.05).

^h^ *q*-value is a False Discovery Rate adjusted p-value (FDR) and it was calculated by Progenesis SameSpots 4.0 software in order to establish how many of the significant ANOVA *p*-values were false positives. Only the protein spots with a *q*-value<0.1 were considered for the mass spectrometry analysis.

^i^ Tukey's post-hoc test was performed on ANOVA *p*-values by GraphPad Prism 6.0 software (^*^p<0.05), (^**^p<0.01), (^***^p<0.001), (^****^p<0.0001), (ns= not significant).

^j^ Average ratio was calculated by GraphPad Prism 6.0 software. It is the ratio between the mean normalized spot volumes of A2780 control cells (CTR), A2780 cells treated with Au(NHC) and A2780 cells treated with Au(NHC)_2_. (Volume e=integration of the optical density over the spot area; %V = V single spot/V total spots included in the reference gel by Progenesis SameSpots 4.0 software).

^k^ Protein spots with a Mascot PMF score ≤70. These spots were subjected to MS/MS analysis to confirm the PMF identification. The results are reported in Supplementary Table 2.

The 51 statistically different expressed protein spots were subjected to Peptide Mass Fingerprint (PMF) analysis using MALDI TOF/TOF mass spectrometer and only those identifications with Mascot PMF score >56 were considered significant (*p*-value<0.05). Such protein spots are highlighted with circles and numbers in the representative gels shown in Figure 4A. The results of PMF identification, together with the statistical analysis data, are reported in Table 2. When MS identification was successful, but with a low Mascot PMF score (≤70), the protein identification was confirmed by MALDI MS/MS analysis. The MS/MS results are summarized in Supplementary Table 1, in which the spot numbers correlate with those reported in Figure 4A and in Table 2.

As reported in Table 2, the experimental isoelectric point (pI) and molecular mass (Mr) values of the 51 identified proteins mostly matched with those theoretically predicted from the genome sequence. In some instances, the same protein was identified in spots that displayed a strong discrepancy between the observed position on 2-DE gels and the normal Mr, with a shift toward lower mass (*e.g.* spots 77, 44, 27, 28, 46, 67) suggesting the presence of protein fragmentation (Table 2; Figure 4A).

**Figure 5:**
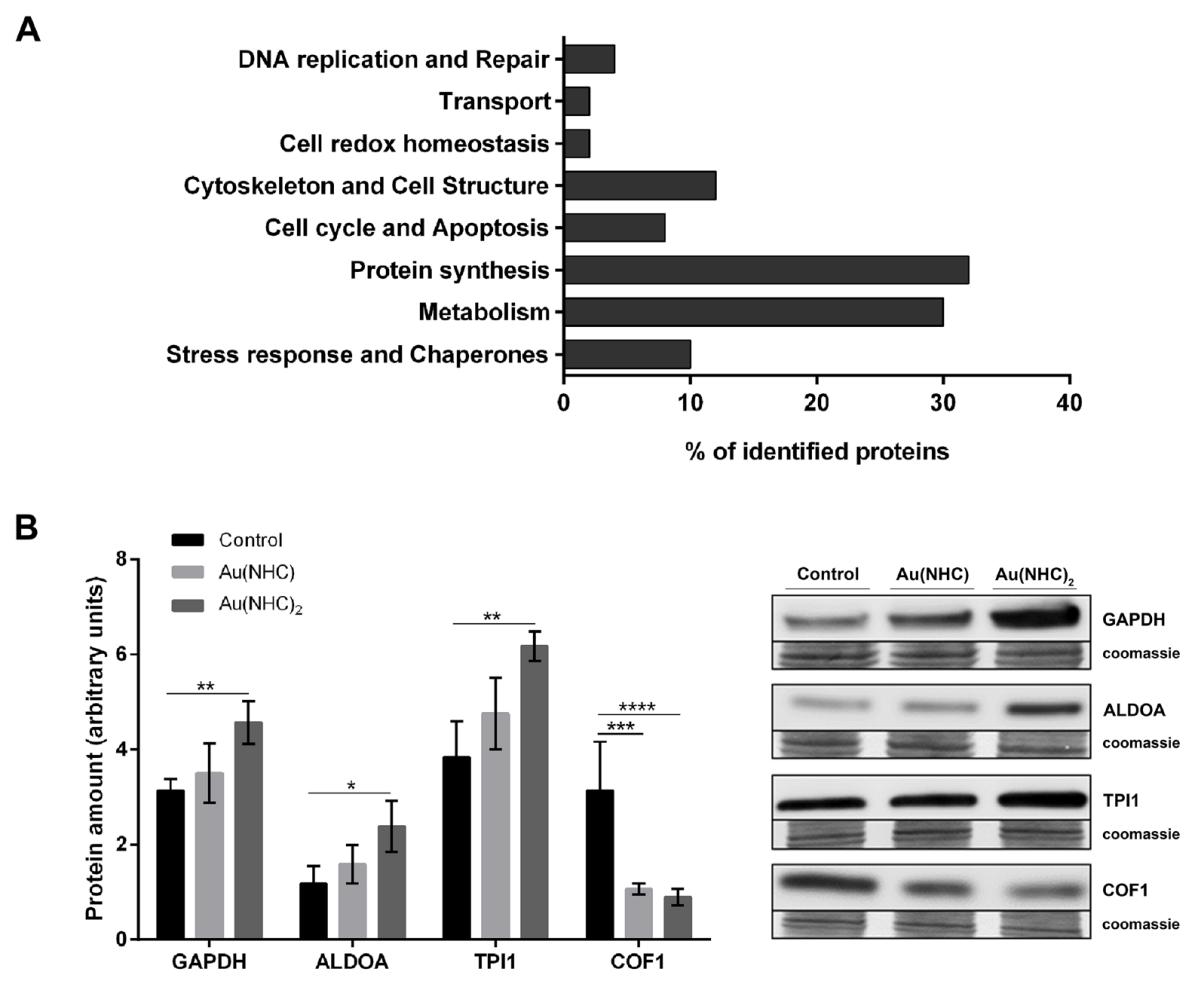
Functional classification of the identified proteins and validation of proteomic results **(A)** The identified proteins were classified based on the Gene Ontology (GO) terms related to their major biological functions using UniProtKB database (http://www.uniprot.org/). Several proteins were associated with more than one function, and in such case, one category was chosen arbitrarily. **(B)** Validation of some identified proteins belonging to the most represented GO categories (metabolism; cytoskeleton and cell structure). Western blot analysis of the glycolytic enzymes GAPDH, ALDOA, TPI1 and the actin-modulating protein COF1 in A2780 cells upon 24 h-exposure to Au(NHC) and Au(NHC)_2_. Representative Immunoblots are shown together with the corresponding Coomassie-stained PVDF membranes. Histogram reports normalized mean relative-integrated-density ± SD values of the GAPDH, ALDOA, TPI1 and COF1 bands. The statistical analysis was carried out using one-way ANOVA test followed by Tuckey's multiple comparisons test using Graphpad Prism v 6.0 (^*^p<0.05, ^**^p<0.01, ^***^p<0.001, ^****^p<0.0001).

The 51 identified proteins were classified into functional categories based on the Gene Ontology (GO) terms related to their major biological functions using UniProtKB database, v. 2017_02 released (http://www.uniprot.org/). When proteins were associated with more than one function, one category was chosen arbitrarily. The most represented classes were those of protein synthesis (32%), metabolism (30%), cytoskeleton and cell structure (12%), stress response and chaperones (10%). The smaller functional classes are cell cycle and apoptosis (8%), DNA replication and repair (4%), cell redox homeostasis (2%) and transport (2%) (Figure 5A). To validate the proteomic result, we focused on proteins belonging to the most represented GO categories such as metabolism and cytoskeleton and cell structure. We measured, by western blot, the protein level of the glycolytic enzymes glyceraldehyde 3-phosphate dehydrogenase (GAPDH), aldolase A (ALDOA), triose phosphate isomerase1 (TPI1) and, the actin-modulating protein cofilin-1 (COF1). Consistent with 2-DE result, the protein level of GAPDH, ALDOA and TPI1 was higher in A2780 cells upon 24 h exposure to Au(NHC)_2_ than in both controls and Au(NHC)-treated cells (Figure 5B). Likewise, the amount of COF1 resulted decreased both in mono-and in bis(carbene) treated cells (Figure 5B).

### Overrepresentation enrichment analysis (ORA)

To provide insight into the cellular changes determined by Au(NHC), and Au(NHC)_2_ in A2780 human ovarian cancer cells, an overrepresentation enrichment analysis (ORA) of GO terms and pathways was carried out on the dataset generated from the identified proteins, using the web-accessible tool WebGestalt v. 2017 (WEB-based Gene SeT AnaLysis Toolket) (http://www.webgestalt.org). This method assesses the statistical overrepresentation of a user-defined, pre-selected gene/protein list of interest in a reference list of known gene/protein sets using a statistical test, *e.g.* the one-sided Fisher's exact test or the hypergeometric distribution followed by multiple test adjustment using Benjamini-Hochberg correction (BH) [46–48]. Table 3 displays the statistically overrepresented GO terms and pathways (*p*-value≤ 0.1 after BH). A detailed composition and statistical parameters of each overrepresented GO term were reported in Supplementary Table 2. The GO Biological Processes (BP), enriched by the 51 identified proteins included ontologies involved in regulation of gene expression, nucleoside metabolic processes and in glycosyl compound metabolic process. We found also enriched the BP term “anatomical structure homeostasis” that indicates a homeostatic process involved in the maintenance of an internal steady-state of an organism, including control of cellular proliferation, death and control of metabolic function. Most of the top score Molecular Function (MF) GO terms belong to protein binding involved in cell adhesion, unfolded proteins, a ubiquitin-like protein ligase, RNA and telomeric DNA. Concerning Cellular Component (CC) GO terms, we found overrepresented the ontologies mitochondrial matrix, cell-cell adherens junction and spliceosomal complex. The functional pathway analysis pointed out the enrichment of carbon metabolism and amino acid biosynthesis pathway, using KEGG database (www.kegg.jp), and of the pathway glycolysis using Panther database (www.pantherdb.org) (Table 3).

**Table 3:** Overrepresentation Enrichment Analysis (ORA) of GO terms and pathways obtained from the identified protein list, using Webgestalt functional enrichment analysis web tool

Gene set	Description	C^a^	O^b^	E^c^	R^d^	*p-*value^e^	FDR^f^
***Biological Process***							
GO:0010608	posttranscriptional regulation of gene expression	454	10	1.35	7.40	6.29E-07	4.79E-04
GO:0034248	regulation of cellular amide metabolic process	359	8	1.07	7.49	9.00E-06	3.43E-03
GO:0071897	DNA biosynthetic process	194	6	0.58	10.4	2.17E-05	5.52E-03
GO:0009123	nucleoside monophosphate metabolic process	308	7	0.92	7.64	3.09E-05	5.89E-03
GO:0006091	generation of precursor metabolites and energy	365	7	1.09	6.44	9.04E-05	1.38E-02
GO:0046939	nucleotide phosphorylation	90	4	0.27	14.9	1.44E-04	1.83E-02
GO:0009141	nucleoside triphosphate metabolic process	290	6	0.86	6.95	2.01E-04	2.08E-02
GO:1901657	glycosyl compound metabolic process	421	7	1.25	5.58	2.19E-04	2.08E-02
GO:0009132	nucleoside diphosphate metabolic process	109	4	0.32	12.3	3.01E-04	2.55E-02
GO:0060249	anatomical structure homeostasis	341	6	1.02	5.91	4.80E-04	3.66E-02
***Molecular Function***							
GO:0098631	protein binding involved in cell adhesion	293	7	0.99	7.00	5.27E-05	1.26E-02
GO:0050839	cell adhesion molecule binding	445	8	1.52	5.27	1.08E-04	1.26E-02
GO:0042162	telomeric DNA binding	30	3	0.10	29.3	1.40E-04	1.26E-02
GO:0051082	unfolded protein binding	105	4	0.36	11.2	4.35E-04	2.92E-02
GO:0003727	single-stranded RNA binding	70	3	0.24	12.6	1.72E-03	9.08E-02
GO:0017025	TBP-class protein binding	21	2	0.07	27.9	2.29E-03	9.08E-02
GO:0003729	mRNA binding	170	4	0.58	6.89	2.60E-03	9.08E-02
GO:0044389	ubiquitin-like protein ligase binding	285	5	0.97	5.14	2.70E-03	9.08E-02
GO:0042287	MHC protein binding	25	2	0.08	23.4	3.24E-03	9.43E-02
GO:0036002	pre-mRNA binding	26	2	0.08	22.5	3.51E-03	9.43E-02
***Cellular Component***							
GO:0043209	myelin sheath	165	7	0.62	11.3	2.11E-06	3.12E-04
GO:0005759	mitochondrial matrix	423	8	1.59	5.04	1.33E-04	7.27E-03
GO:0005913	cell-cell adherens junction	318	7	1.19	5.87	1.47E-04	7.28E-03
GO:0009295	nucleoid	45	3	0.17	17.8	6.16E-04	2.28E-02
GO:0005681	spliceosomal complex	173	4	0.65	6.16	3.81E-03	1.00E-01
***Pathways (KEGG database)***							
hsa01200	Carbon metabolism	114	5	0.49	10.2	1.13E-04	3.43E-02
hsa01230	Biosynthesis of amino acids	75	4	0.32	12.3	2.80E-04	4.24E-02
***Pathways (Panther database)***							
P00024	Glycolysis	17	3	0.08	36.5	5.50E-05	6.22E-03

^a^ The number of reference genes in the category

^b^ The number of genes in the user gene list and also in the category

^c^ The expected number in the category

^d^ Ratio of enrichment

^e^ *p*-value from hyergeometric test

^f^ FDR from Benjamini and Hochberg (BH).

### Influence of gold carbene complexes on cancer cell metabolism

**Figure 6:**
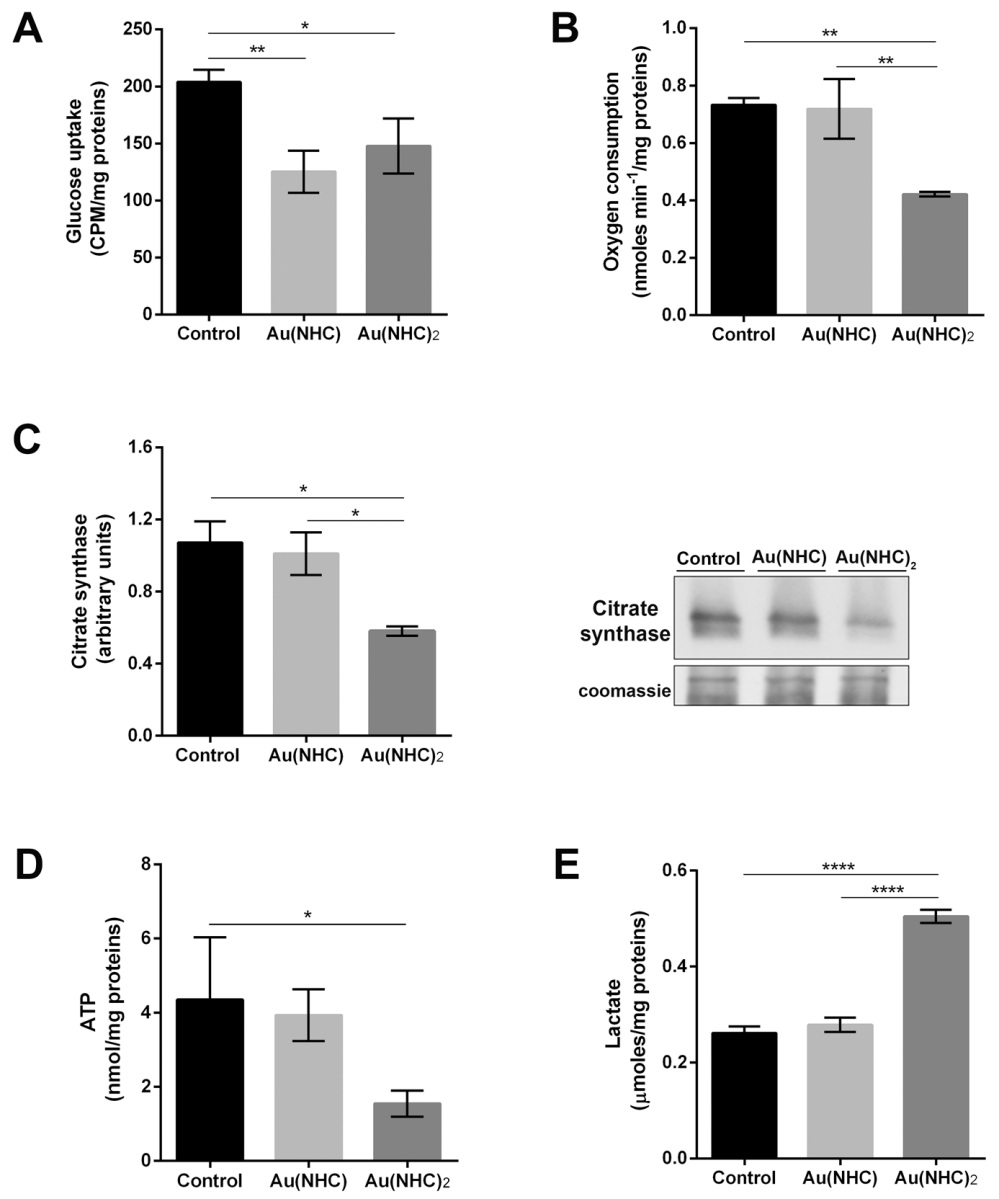
Influence of gold carbene complexes on cancer A2780 cell metabolism A2780 cells were treated with Au(NHC) and Au(NHC)_2_ for 48 h with their 72 h-exposure IC_50_-dose **(A)** Glucose uptake was assessed using 1μCi 2-deoxy-D-2-3H-glucose (1mCi/0.1 mmol) as described, in detail, in Materials and Methods section. **(B)** Oxygen consumption was measured using a Clark-type O_2_ electrode from Hansatech. **(C)** Western blot analysis of citrate synthase (CS). Representative Immunoblots are shown together with the corresponding Coomassie-stained PVDF membranes. Histogram reports normalized mean relative-integrated-density ± SD values of the CS bands. **(D)** Lactate amount was detected in one millilitre of medium supernatant using a commercial L-lactic acid Assay Kit (Megazyme). **(E)** ATP amount was assessed with ATP Determination Kit (Molecular Probes) according to manufacturing instruction. Histograms report the mean values ±SD of at least three independent experiments. The statistical analysis was carried out using one-way ANOVA test followed by Tuckey's multiple comparisons test using Graphpad Prism v 6.0 (^*^p<0.05, ^**^p<0.01, ^****^p<0.0001).

Proteomic and bioinformatic data highlighted a pronounced cytotoxic effect mostly by Au(NHC)_2_ on cell energy metabolism. To strengthen these data, we evaluated the ability of these compounds to affect cellular glycolytic and mitochondrial activities. Therefore, we carried out an assessment of: i) glucose uptake, ii) oxygen consumption, iii) citrate synthase levels, iv) ATP levels and v) lactate production. A2780 cells were treated for both 24 and 48 h with mono or bis(carbene) at a concentration corresponding to their 72 h-exposure IC_50_-dose. In the first 24 h of treatment, there were no significant differences between carbene-treated and untreated cells (data not shown). After 48 h, all four parameters showed an evident change only in Au(NHC)_2_-treated cells whereas monocarbene was slightly less effective. Even if, both gold complexes gave rise to a significant decrease of glucose uptake (Figure 6A), only the bis(carbene) turned out to be an inhibitor of respiration. Indeed, in Au(NHC)_2_-treated cells the oxygen consumption diminished of 1.7-fold in comparison with Au(NHC) and control cells (Figure 6B). Remarkably, upon Au(NHC)_2_ exposure, the amount of citrate synthase (CS), the rate-limiting enzyme of TCA cycle, was lower compared with control cells, showing a decrease of about 2-fold (Figure 6C). In line with the observed slowdown of mitochondrial respiration, the ATP level was reduced of about 2.8-fold (Figure 6D); conversely, the lactate production was increased of about 1.9-fold in bis(carbene)-treated cells (Figure 6E).

### Interference with cellular redox homeostasis

**Figure 7:**
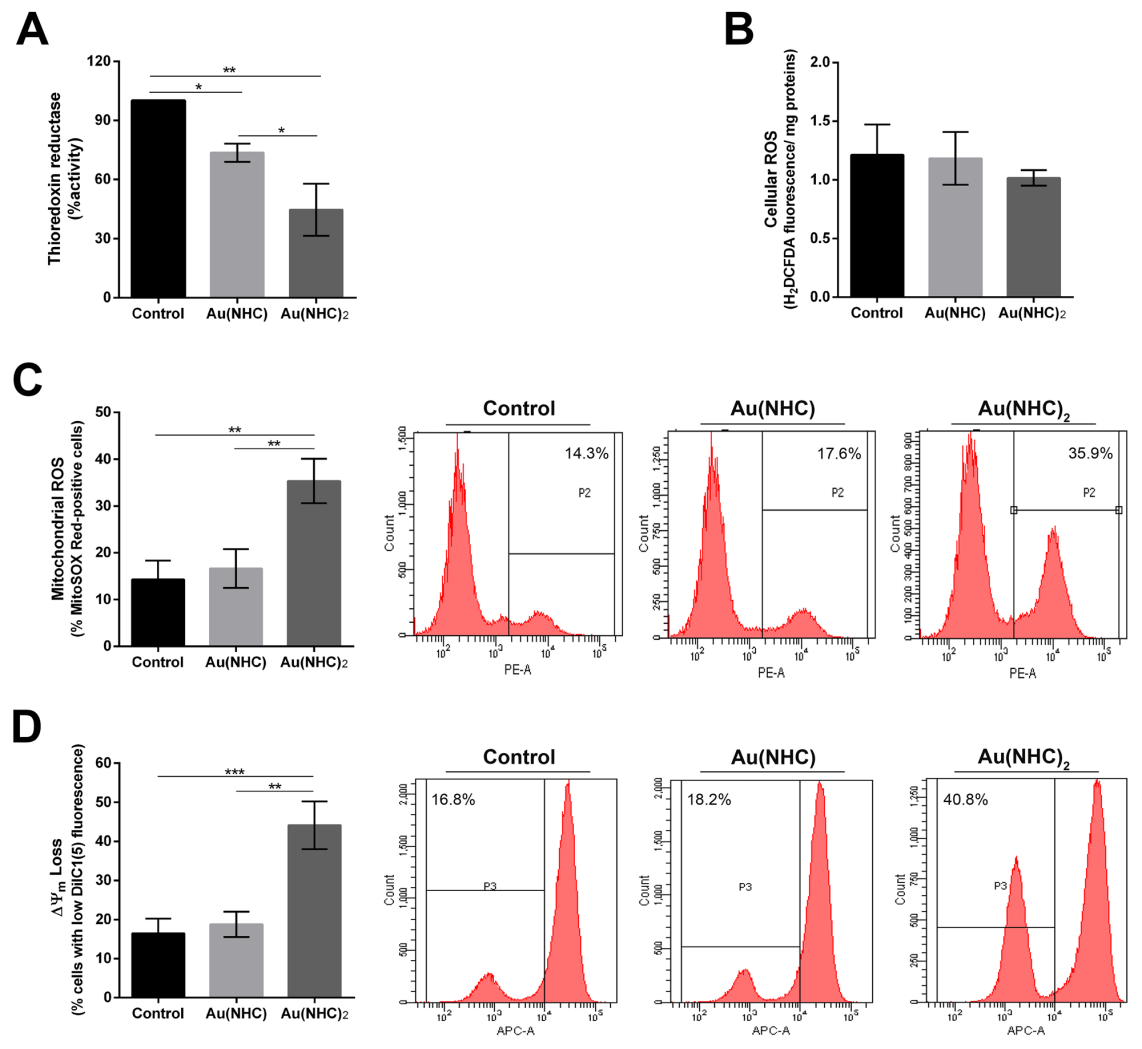
Interference with cellular redox state in A2780 cells A2780 cells were treated with Au(NHC) and Au(NHC)_2_-72 h-IC_50_-dose. **(A)** TrxR enzyme inhibition assay was performed after 24 h of treatment using a commercial thioredoxin reductase assay kit (Sigma-Aldrich). **(B)** Cellular generic ROS production were evaluated after 72 h of treatment by the fluorogenic dye H_2_DCFDA analysed using a fluorescence spectrophotometer. **(C)** Mitochondrial superoxide anion production was assessed using the fluorogenic dye MitoSOX Red production by flow cytometry after 72 h of treatment. **(D)** Mitochondrial membrane potential (Δψ_m_) using the cyanine dye DiIC1(5) and flow cytometry, after 72 h of treatment. Flow cytometric images are representative of three independent experiments. Histograms report the mean values ±SD. The statistical analysis was carried out using one-way ANOVA test followed by Tuckey's multiple comparisons test using Graphpad Prism v 6.0 (^*^p<0.05, ^**^p<0.01, ^***^p<0.001).

Mitochondria and the oxidative phosphorylation pathway have already been identified as primary cellular targets for auranofin and several NHC gold complexes through inhibition of the seleno-enzyme thioredoxin reductase (TrxR) [26, 35–37, 40]. Indeed, TrxR is an essential antioxidant enzyme for maintaining the intracellular redox homeostasis and is involved in cell growth and survival. Its inhibition leads to an increase of cellular ROS, an impairment of mitochondrial functions, and finally to apoptosis [26]. Within this frame, we evaluated whether the antiproliferative effects of Au(NHC) and Au(NHC)_2_ complexes in A2780 cells could be explained by direct inhibition of TrxR. The enzyme activity was assessed on protein extracts obtained from A2780 cells treated, for 24 h, with each carbene complex IC_50_-dose. Notably, both gold carbenes inhibited TrxR (Figure 7A). The most effective compound was Au(NHC)_2_ causing about 50 percent inhibition of enzyme activity, whereas Au(NHC) caused a smaller reduction (ca. 25 percent). On the ground of these results, we evaluated whether the inhibitory effect on TrxR could lead to ROS formation and changes of the mitochondrial membrane potential (Δψ_m_). The influence of carbene complexes on the cellular reactive oxygen species (ROS) levels were investigated in time course experiments (12, 24, 48 and 72 h after treatment) using the fluorogenic dye H_2_DCFDA in conjunction with flow cytometer. In this case, both drugs were unable to rise cellular ROS. Figure 7B shows the results 72 h after treatment. We further assessed the production of the superoxide anion, the predominant ROS species in mitochondria, using the fluorogenic dye MitoSOX Red specifically targeted to mitochondria, and flow-cytometry. We performed the same time course experiments and we observed that only Au(NHC)_2_ led to a 2.5-fold increase of mitochondrial ROS production after 72 h of treatment (Figure 7C). Since increase of mitochondrial ROS may be initiated by dysfunctional mitochondria [49], we analysed by flow-cytometry the mitochondrial membrane potential (Δψ_m_), on the same treated-cells, using the cyanine dye DiIC1(5) (Figure 7D). In line with the data on mitochondrial superoxide anion levels, this experiment pointed out that only Au(NHC)_2_ affected the Δψ_m_ and after 72 h of exposure. Indeed, we observed in Au(NHC)_2_ a 2.8-fold increase of cells with lower Δψ_m_ values in comparison with both control and monocarbene-treated cells.

### Au(NHC) and Au(NHC)_2_ induce antiproliferative effects in other human ovarian cancer cell lines

**Figure 8:**
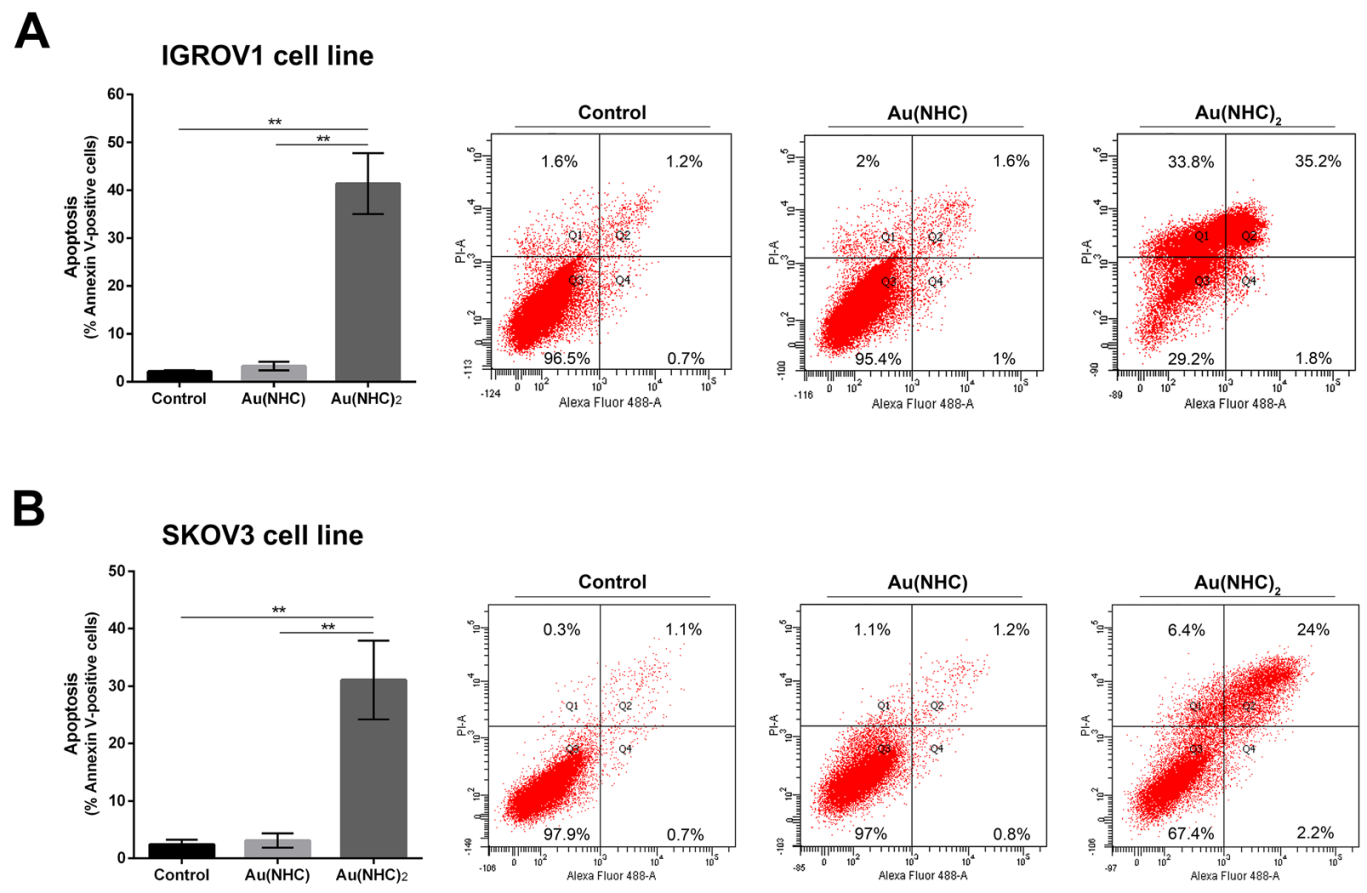
Apoptosis induction in IGROV1 and SKOV3 ovarian cancer cells Percentage of apoptotic cells shown by flow cytometry analysis of annexin V/propidium iodide-stained IGROV1 **(A)** and SKOV3 **(B)** cells treated for 72 h with Au(NHC) and Au(NHC)_2_-72 h-exposure IC_50_-dose. Flow cytometric images are representative of three independent experiments. Histograms report the mean values ±SD. The statistical analysis was carried out using one-way ANOVA test followed by Tuckey's multiple comparisons test using Graphpad Prism v 6.0 (^**^p<0.01).

To strengthen the data achieved up to now on carbene antiproliferative mechanisms in A2780 cells we carried out confirmatory experiments on other human ovarian cancer cell lines. We tested IGROV1 and SKOV3 established human ovarian cancer cell lines that constitute relevant *in vitro* tumor models, for ovarian cancer research. IGROV1 cell line was established from an ovarian endometrioid adenocarcinoma, whereas SKOV3 cell line derived from ascitic fluid from a caucasian female with an ovarian tumour. As confirmatory experiments, we decided to evaluate: i) the cytotoxicity of the two gold carbenes against the two above mentioned tumor cell lines and the apoptotic cell death, ii) their influence on cell metabolism and, iii) the inhibitory potential toward TrxR. The cytotoxic effects of these gold(I) complexes were evaluated on IGROV1 and SKOV3 cell lines by the MTT proliferation assay and compared with those of cisplatin (Table 4). After a 72h exposure, both carbenes were more active than cisplatin (6.1 μM) against SKOV3 cells although Au(NHC)_2_ was highly more cytotoxic (0.5 μM) than Au(NHC) (4.7 μM). As far as the IGROV1 cell line is concerned, the following cytotoxic activity was observed: Au(NHC)_2_ > cisplatin > Au(NHC) (*i.e.* ca. 0.4, 1.2, 3.9 μM, respectively). Thus, different antiproliferative effects of the study gold carbenes on SKOV3 and IGROV1 are in agreement with findings achieved in A2780 cells. Afterwards, we examined if Au(NHC) and Au(NHC)_2_ also lead to apoptotic cell death in IGROV1 and SKOV3 cells. We performed flow cytometry analysis of annexin V/propidium iodide-stained cells treated with Au(NHC) or Au(NHC)_2_ 72 h- IC_50_-dose, for 72 h. Surprisingly, the cell-state distribution highlighted a pronounced antiproliferative effect in both cell line only by Au(NHC)_2_. As shown in Figure 8, about 40% of late apoptotic IGROV1 cells (Figure 8A) and about 30% SKOV3 cells (Figure 8B) were present in bis(carbene)-treated cells. The monocarbene-treated cells behaved like controls (Figure 8A and 8B).

**Table 4:** Antiproliferative effects of Au(NHC), Au(NHC)_2_ and cisplatin on cell growth of SKOV3 and IGROV1 ovarian cancer cells after 72 h drug exposure

	IC_50_ (μM) ± SD		
	Au(NHC)	Au(NHC)_2_	Cisplatin
**SKOV3**	4.708±0.842	0.552±0.044	6.111±1.098
*n*	*3*	*3*	*3*
**IGROV1**	3.922±0.533	0.387±0.057	1.170±0.177
*n*	*3*	*3*	*3*

n, number of independent experiments; SD, standard deviation.

**Figure 9:**
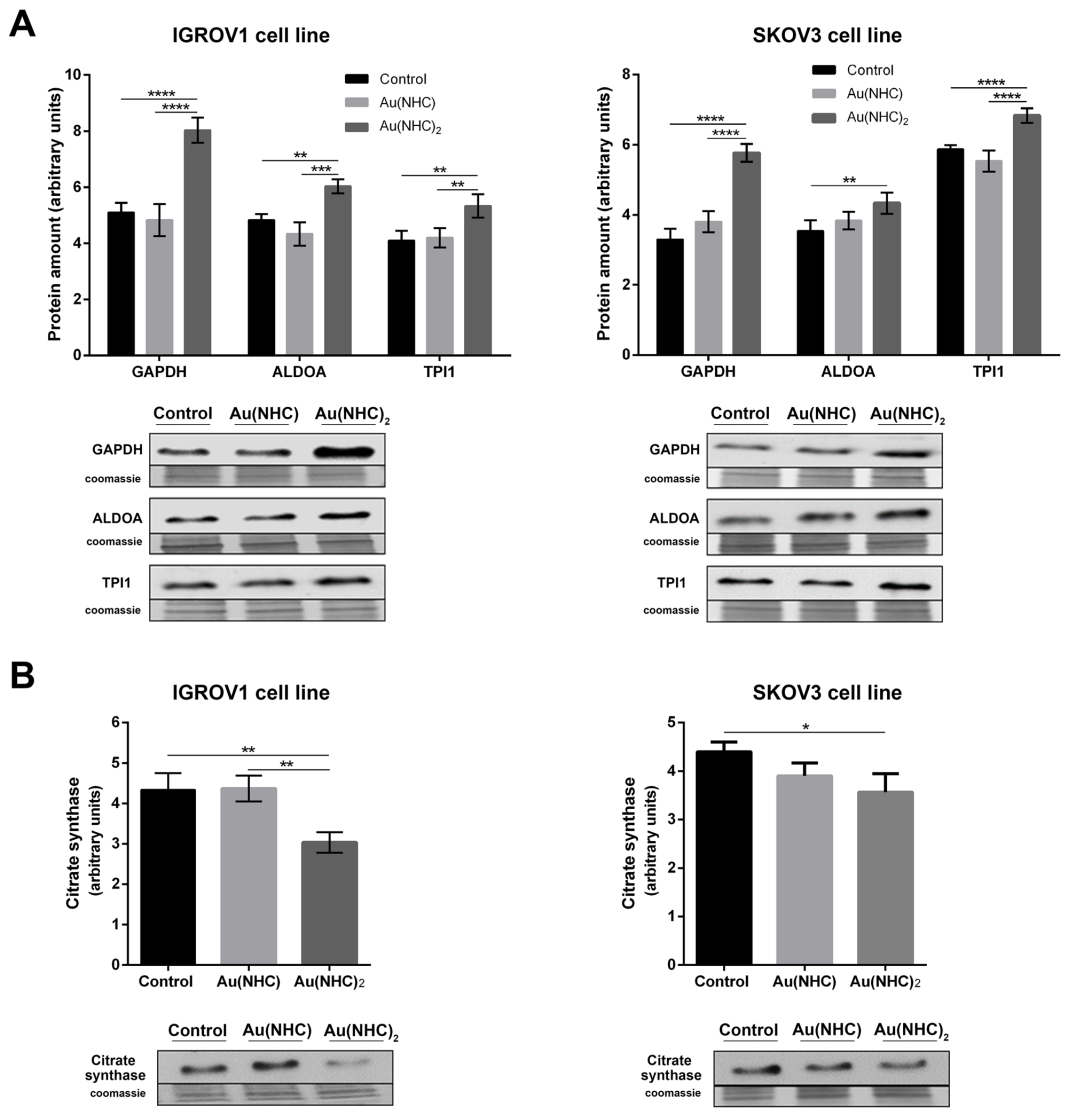
Effects of carbenes on the level of selected glycolytic and mitochondrial enzymes in IGROV1 and SKOV3 cells Western blot analysis of the glycolytic enzymes GAPDH, ALDOA, TPI1 **(A)** and of the mitochondrial enzyme CS **(B)** in IGROV1 and in SKOV3 cells upon exposure to Au(NHC) and Au(NHC)_2_. Representative Immunoblots are shown together with the corresponding Coomassie-stained PVDF membranes. Histogram reports normalized mean relative-integrated-density ± SD values of the GAPDH, ALDOA, TPI1 and CS bands. The statistical analysis was carried out using one-way ANOVA test followed by Tuckey's multiple comparisons test using Graphpad Prism v 6.0 (^*^p<0.05, ^**^p<0.01, ^***^p<0.001, ^****^p<0.0001).

**Figure 10:**
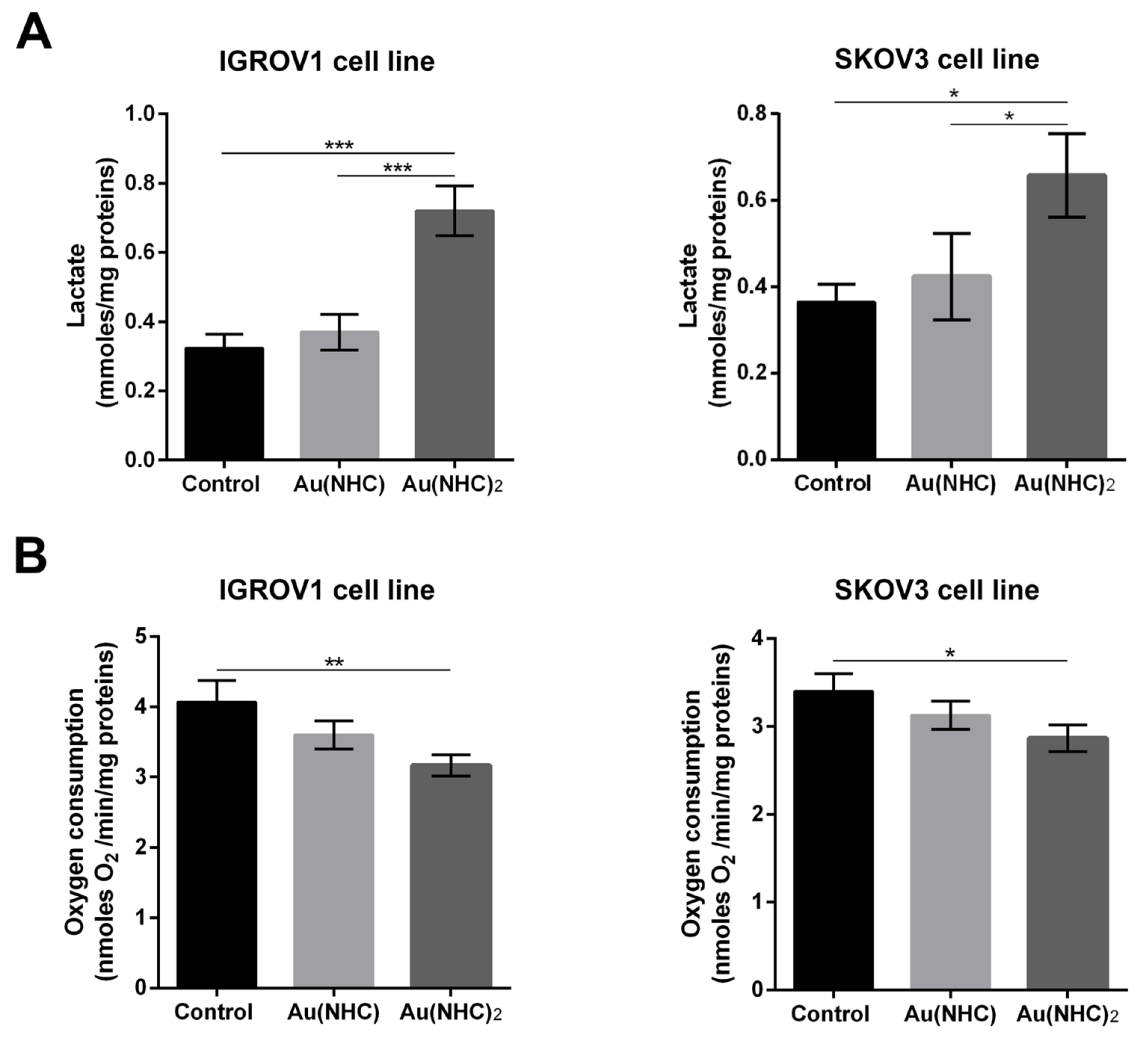
Influence of gold carbene complexes on cancer IGROV1 and SKOV3 cell metabolism IGROV1 and SKOV3 cells were treated with Au(NHC) and Au(NHC)_2_ for 48 h with their 72 h-exposure IC_50_-dose **(A)** Lactate amount was detected in one millilitre of medium supernatant using a commercial L-lactic acid Assay Kit (Megazyme); **(B)** Oxygen consumption was measured using a Clark-type O_2_ electrode from Hansatech. (For details, see Materials and Methods). Histograms report the mean values ±SD of at least three independent experiments. The statistical analysis was carried out using one-way ANOVA test followed by Tuckey's multiple comparisons test using Graphpad Prism v 6.0 (^*^p<0.05, ^**^p<0.01, ^***^p<0.001).

To evaluate the influence of Au(NHC) and Au(NHC)_2_ on IGROV1 and SKOV3 cellular glycolytic and mitochondrial metabolism, we analysed: i) the level of some glycolytic enzymes pointed out by proteomic analysis on A2780 cells, ii) the level of the citrate synthase, iii) the lactate production and iv) the oxygen consumption. Cells were treated for both 24 and 48 h with the mono or bis(carbene) 72 h-exposure IC_50_-dose. In line with the data on A2780 cells, in the first 24 h of treatment, we detected a significant change only in the glycolytic enzyme levels (Figure 9A), whereas all other parameters showed an evident difference just after 48 h of treatment (Figure 9B and Figure 10). Specifically, the western blot analysis on the glycolytic enzymes GAPDH, ALDOA and TPI1 highlighted an increased level only in bis(carbene) IGROV1 and SKOV3-treated cells (Figure 9A). Besides, only Au(NHC)_2_ gave rise to a decrease in the amount of the TCA cycle key enzyme CS (Figure 9B). We found a decrease of about 1.4-fold in IGROV1 cells and of about 1.2-fold in SKOV3 cells. In any case the behaviour of Au(NHC)-treated cells was similar to controls (Figure 9). Consistent with the increment of glycolytic enzyme level, we found that the lactate production was increased in bis(carbene)-treated cells (Figure 10A). In IGROV1, we detected a lactate production of about 2-fold higher respect to controls and in SKOV3 of about 1.8-fold (Figure 10A). This enhancement in lactate production was accompanied in Au(NHC)_2_-treated cells by a slight slowdown of oxygen consumption. Indeed, in IGROV1 cells we observed a respiration reduction of about 1.3-fold respect to controls and in SKOV3 of about 1.2-fold (Figure 10B). Overall, the monocarbene complex did not lead to significant changes in both lactate production and oxygen consumption (Figure 10A and 10B).

**Figure 11:**
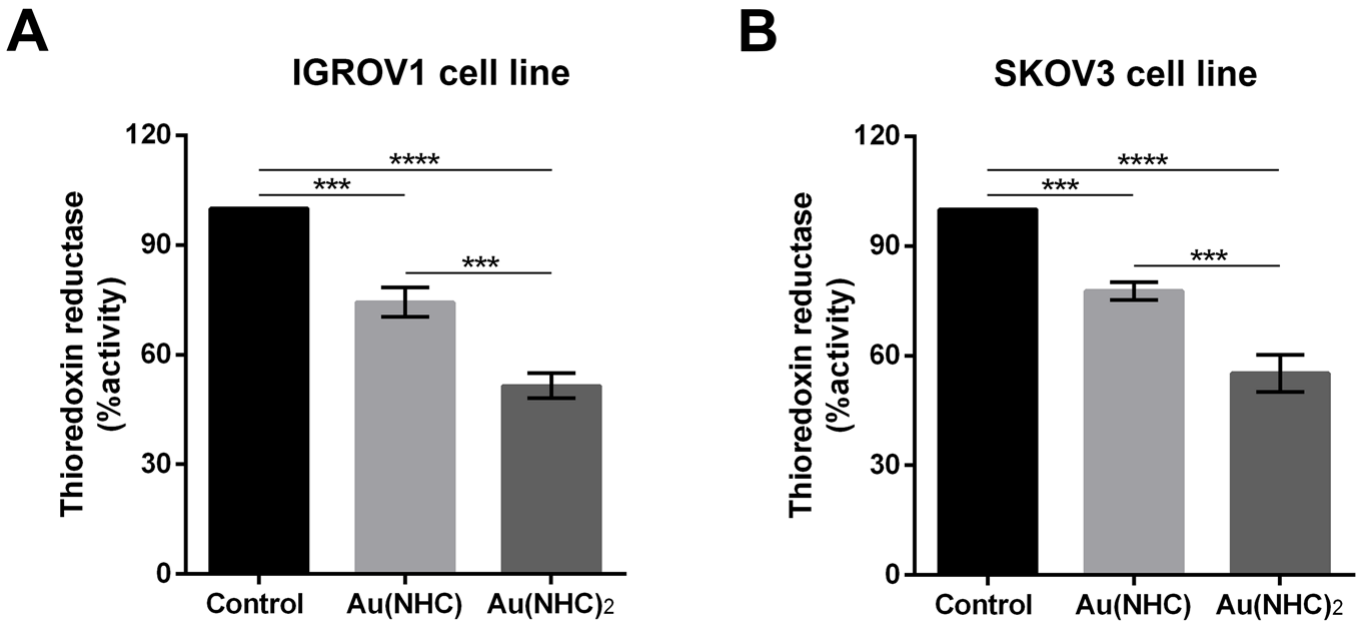
Inhibition of TrxR by carbenes in IGROV1 and SKOV3 cells IGROV1 and SKOV3 cells were treated with Au(NHC) and Au(NHC)_2_-72 h-IC_50_-dose. TrxR enzyme inhibition assay was performed after 24 h of treatment, using a commercial thioredoxin reductase assay kit (Sigma-Aldrich), in **(A)** IGROV1 and **(B)** SKOV3 cells. For details, see Materials and Methods. Histograms report the mean values ±SD. The statistical analysis was carried out using one-way ANOVA test followed by Tuckey's multiple comparisons test using Graphpad Prism v 6.0 (^***^p<0.001, ^****^p<0.0001).

Even if, only the bis(carbene) complex gave rise to significant changes in cell metabolism, both Au(NHC) and Au(NHC)_2_ turned out to be inhibitors of TrxR activity (Figure 11). The TrxR enzymatic assay pointed out that after 24 h of Au(NHC) treatment the enzyme activity was reduced of about 25% in IGROV1 cells and about 23% in SKOV3. The bis(carbene) showed a greater inhibitory potential triggering a reduction in TrxR activity of about 48% in IGROV cells and of about 44% in SKOV3 cells (Figure 11).

## DISCUSSION

### Cytotoxicity, logP, cell cycle alterations and cell death

In the present work, we sought to investigate the cytotoxic effects and the associated proteomic alterations of two new gold(I)-N-heterocyclic carbene complexes, namely Au(NHC) and Au(NHC)_2_, in A2780 human ovarian cancer cells. Valuable antiproliferative effects were observed for both carbene complexes, but to different degrees, with Au(NHC)_2_ being more active than the monocarbene. This higher cytotoxicity of the bis(carbene) was accompanied by a cell cycle arrest with a significant accumulation of cells in the G_1_ phase whereas the monocarbene did not induce any specific phase arrest. However, both mono and bis(carbene) treatments led to cell death via apoptosis. In determining whether caspase cascade was involved the in cell death process, we observed that both Au(NHC) and Au(NHC)_2_ triggered a significant activation of caspase-8 whereas caspase-9 and caspase-3 displayed an increase only with the bis(carbene). Caspase-8 and caspase-9 are initiator caspases of the extrinsic and the intrinsic apoptotic pathway, respectively. These data indicated, for the monocarbene, a cell death mediated by the extrinsic apoptotic pathway and, for the bis(carbene) an involvement of both extrinsic and intrinsic apoptotic pathways. It is noteworthy that, besides the lower lipophilicity compared to monocarbene, the bis(carbene) displayed a greater cytotoxicity and, properties like those observed in delocalized lipophilic cations (DLCs) [50], could be expected for Au(NHC)_2_. As a matter of fact, in the scientific literature several examples of charged complexes as DLCs have been reported that are able to accumulate inside mitochondria, penetrating the hydrophobic barrier of the cellular membranes due to the electric gradient between membranes’ inner and outer layers [51]. All these findings suggested, in terms of structure–activity relationships, that the introduction of the second carbene ligand in Au(NHC)_2_ enhanced the antiproliferative effects of gold NHC center [52, 53]. This trend of increasing cytotoxicity when substituting a chloride with a phosphane or NHC ligand has previously been observed for other gold (I)-NHC complexes [35, 54]. In part, this has been associated to the formation of cationic species [Au(NHC)_2_]^+^ which might induce mitochondrial accumulation and improve cytotoxic effects [32, 55, 56].

### Proteomic results and affected proteins

To identify proteins involved in the antiproliferative effects of the Au(NHC) and Au(NHC)_2_ in A2780 cells, a comparative proteome study using 2-DE and MS based analysis has been carried out. The results pointed out 51 differentially expressed proteins. In line with the cytotoxic properties of the two gold carbene compounds, the number of modulated protein spots was higher in Au(NHC)_2_-treated cells and lower in Au(NHC)-treated cells. It is noteworthy that the 19 modulated proteins highlighted in monocarbene-treated cells varied with the same trend in bis(carbene)-treated cells. In addition, only six proteins differed between Au(NHC) and Au(NHC)_2_-treated cells, showing the same tendency both in control and in monocarbene-treated cells.

All the 51 modulated proteins play specific roles in a variety of biological processes. Most of them belong to GO functional classes *i.e.* protein synthesis, metabolism, cytoskeleton and cell structure, stress response and chaperones. To understand the biological meaning behind the list of the identified proteins, we analysed them by the functional enrichment analysis web tool WebGestalt. We found statistically enriched the BP term posttranscriptional regulation of gene expression. This GO term included 6 proteins of the hnRNP (heterogeneous nuclear ribonucleoproteins) family that comprises multifunctional proteins participating in a variety of cellular processes related to mRNA splicing, stability and transport to the cytoplasm. Given their central roles in the regulation of gene expression, deregulation of individual hnRNPs was involved in tumour development and progression, including inhibition of apoptosis, angiogenesis and cell invasion [57]. Moreover, hnRNP proteins significantly affect responses to chemotherapy, acting as mediators or modulators of drug-induced apoptosis [58]. However, the pathomechanisms of many hnRNPs remain to be elucidated [59]. In the current study the subtypes Q, D, A2/B1, A1 and an isoform of K were upregulated whilst the subtypes F and another isoform of K were downregulated in bis(carbene) treated cells. The monocarbene affected, with the same trend, only the subtypes D, A2/B1, A1 and an isoform of K. Since products of alternative splicing often possess different, even opposite functions, the change of abundance patterns is likely to play a significant role in the early cellular response to drug treatment [57, 59]. Further investigations are required to strengthen their involvement in carbene antiproliferative effects. In the enriched GO term posttranscriptional regulation of gene expression, we found also the phosphoprotein nucleophosmin (NPM1) mainly localized at nucleoli. This protein belongs to a histone chaperones family, the Nucleophosmin/nucleoplasmin (NPM) family, and has an important role in several pathways including mRNA transport, chromatin remodeling, apoptosis, and regulation of tumor suppressors p53/TP53 and ARF [60]. Nucleophosmin is frequently overexpressed in tumours. Previous studies showed that NPM1 is downregulated in cancer cells during drug-induced apoptosis [61, 62]. Our finding that both carbene complexes induced a downregulation of NPM1, as other anticancer drugs mentioned above, suggested a direct or indirect involvement of this protein in the apoptotic process in A2780 cells.

The functional enrichment analysis highlighted also the overrepresentation of GO BP terms and pathways related to carbon metabolism and glycolysis. These GO categories comprise the glycolytic enzymes GAPDH, ALDOA and TPI1 that were upregulated only in Au(NHC)_2_ treated cells. Conversely, the amount of several enzymes involved in cellular respiration and ATP production decreased with both carbenes (*i.e*. ACO2, AK2, ATP5C1) and some of them only with bis(carbene) (*i.e.* CYP4A22 and VCP). These data suggested a possible effect of Au(NHC)_2_ on cell energy metabolism. To strengthen these data, we evaluated the cellular glycolytic and mitochondrial activity measuring: i) glucose uptake, ii) oxygen consumption, iii) citrate synthase levels, iv) ATP levels and v) lactate production. The obtained results were in line with the high cytotoxic potency of bis(carbene) complex. Indeed, after 48 h, all four parameters showed an evident change in Au(NHC)_2_-treated cells whereas monocarbene was slightly less effective. While, both mono and bis(carbene) complexes induced a significant decrease of glucose uptake, mostly Au(NHC)_2_ elicited a strong inhibition of respiration coupled with a decrease of citrate synthase (CS) amount, the rate-limiting enzyme of TCA cycle. In Au(NHC)_2_-treated cells, this impairment of respiration and the dysfunctional TCA cycle were accompanied by a lower ATP content. The lactate production, in turn, was enhanced, suggesting a glycolysis acceleration. Thus, the decrease of oxygen consumption and the increase of lactate production reflected the ability of Au(NHC)_2_ to impair respiration and to determine a metabolic shift towards glucose fermentation probably as a compensatory mechanism. In this analysis, mitochondria appeared to be a cellular target mostly of bis(carbene) compound.

Among the six differentially expressed proteins between the two carbene complexes, we found two proteins involved in cell metabolism: OXCT1, the key enzyme for ketone body catabolism that increased in bis(carbene)-treated cells and the protein HIBCH involved in the pathway amino-acid degradation that decreased respect to both controls and monocarbene treated cells. We found also two proteins involved in the GO category protein synthesis: the elongation factor 2 (EEF2) identified as a fragment and the zinc finger protein 18, both increased in bis(carbene)-treated cells. Two proteins belong to the GO category cytoskeleton and cell structure such as the vascular cell adhesion protein VCAM1, identified as a fragment that increased after bis(carbene) exposure and the proliferation-associated protein 2G4 (PA2G4) involved in growth regulation that conversely decreased. The meaning of the six differentially expressed proteins between the mono- and bis(carbene) remains to be clarified. In this regard, we must investigate deeply the role of the two gold carbenes in the GO biological processes to which the six proteins belong (protein synthesis, cytoskeleton etc.).

### Thioredoxin reductase as a target

The seleno-enzyme thioredoxin reductase (TrxR) is an essential antioxidant enzyme for maintaining the intracellular redox homeostasis and is involved in cell growth and survival. Its inhibition leads to an increase of cellular ROS, an impairment of mitochondrial functions, and finally to apoptosis. Since TrxR had already been identified as an important target of several gold(I) complexes (*i.e.* auranofin, gold(I) NHC complexes) [26, 35–38], the mono and bis(carbene) complexes were tested for their ability to inhibit TrxR in A2780 treated cells. Both gold compounds gave rise early (after 24 h) to inhibition of TrxR, but the most potent inhibitor was Au(NHC)_2_, correlating well with its higher cytotoxic effects with respect to the monocarbene. On the ground of these results, we evaluated whether the inhibitory effect on TrxR could lead to ROS formation and alteration of the mitochondrial membrane potential (Δψ_m_). Unexpectedly, in both mono and bis(carbene)-treated cells, the TrxR inhibition was not followed by an accumulation of cytosolic ROS, even after 72 h of exposure, indicating that generalised oxidative stress is not responsible for the observed events. However, in bis(carbene) treated cells we found an increase of mitochondrial superoxide associated with a decrease Δψ_m_. These findings further reinforced the view that the mitochondrial pathways are primarily involved in Au(NHC)_2_-dependent proapoptotic effects, most likely in relation to selective inhibition of thioredoxin reductase. On the other hand, a direct involvement of oxidative stress could be ruled out since mitochondrial ROS production and membrane depolarization can be detected only after 72 h, when the apoptosis pathway was already started. Thus, it is likely that ROS initiated from dysfunctional mitochondria instead of being the primary cause of the Au(NHC)_2_ cytotoxic effects. Likewise, Rigobello et *al.* demonstrated that, in Jurkat T cells, gold(I) complexes (auranofin and TepAu) induced apoptosis through inhibition of TrxR but with limited oxidative stress [63].

Thioredoxin is the main substrate of TrxR. Inhibition of TrxR activity led to the decrease of reduced (active) form of thioredoxin triggering an unbalance of cell redox state [64]. It is noteworthy that, besides its direct role in regulating cellular ROS level, thioredoxin controls the redox state of several proteins involved in different redox-signaling pathways. For example, thioredoxin has a direct interaction with the apoptotic pathway through the negative regulator ASK1 as well as through the phosphatase and tensin homolog (PTEN) [65, 66]. Therefore, our data seems to suggest that, instead of a direct role in ROS production, the Au(NHC)_2_ cytotoxicity could mainly affect, through TRxR inhibition, molecular targets involved in redox-signalling pathways.

### Antiproliferative effects in other human ovarian cancer cell lines

Ovarian cancer is a morphologically and biologically heterogeneous disease and therapeutic approaches need to account for inter-patient and intra-tumoural heterogeneity. We have therefore decided to confirm the cytotoxic activity of the two gold carbenes in established cell lines derived from other human ovarian neoplasms such as IGROV1 and SKOV3 cells that are commonly used for *in vitro* studies in ovarian cancer research. The IC_50_ values and the analysis of apoptosis revealed a slightly lower cytotoxicity for both mono and bis(carbene) compounds in these cell lines respect to A2780 cells. However, the greater activity of Au(NHC)_2_ compared to Au(NHC) was confirmed. In fact, only Au(NHC)_2_ triggered apoptotic cell death both in IGROV1 and in SKOV3, with SKOV3 showing more resistance. This trend has also occurred analysing the carbene influence on cell metabolism. The results pointed out that only the bis(carbene) led to enhancement of lactate production coupled with a slowdown of respiration in both IGROV1 and SKOV3 cell lines. These data were also supported by western blot analysis on both glycolytic and mitochondrial enzymes. Indeed, only the bis(carbene) provided an increase of the glycolytic enzymes GAPDH, ALDOA and TIP1 and decrease of the mitochondrial enzyme CS. Once again, the Au(NHC)_2_ effect in IGROV1 cells was in line with that in A2780 cells, whereas in SKOV3 cells it was less effective. Concerning the inhibitory potential toward TrxR the results were in line with those seen in A2780 cells. Indeed, both mono and bis(carbene) inhibit the enzyme activity in IGROV1 and SKOV3.

All these findings revealed that regardless of the cell lines, the bis(carbene) owns a cytotoxicity and an inhibitory capacity of TrxR greater than the monocarbene. On the other hand, the degree of antiproliferative and metabolic effects of the Au(NHC)_2_ differs depending on the cell line. A2780 and IGROV1 showed a very similar sensitivity towards Au(NHC)_2_, whereas SKOV3 appeared slightly more resistant. Thus, it is possible to suggest that the mechanism of action of the two carbenes may vary in part according to the cell lines. To better understand the mechanism of action of two carbenes and their potentialities as anticancer drugs we will extend our studies to further human cancer cell lines.

### Concluding remarks

In conclusion, upon comparing the effects of these two gold carbene complexes through all the experiments with those of control cells, it clearly emerged that Au(NHC)_2_ is a far more effective compound versus the three cancer cell lines used in this study. Its behaviour is consistent with that of other delocalized lipophilic cations (DLCs) [54]. DLCs have been investigated as a new approach to cancer chemotherapy, which exploits their selective accumulation in mitochondria of cancer cells because of the elevated mitochondrial membrane potential that is a shared feature for many tumor cell lines.

Notably, Au(NHC)_2_ treatment leads to strong antiproliferative effects, potent inhibition of TrxR activity, decrease of mitochondrial respiration coupled with a lower mitochondrial membrane potential and higher glycolytic activity followed by a decrease of ATP level and finally cell death. Based on these evidences, both at the molecular and cellular level, we propose that the relevant cytotoxic actions produced by Au(NHC)_2_ are mainly the result of potent inhibition of thioredoxin reductase; the alterations of mitochondrial functions, elicited by profound TrxR inhibition, would lead to cell apoptosis. However, the exact identification of all the steps connecting the inhibition of TrxR to programmed cell death is not achieved yet and requires further studies.

## MATERIALS AND METHODS

### Materials

RPMI 1640 cell culture medium, fetal calf serum (FCS), and phosphate-buffered saline were obtained from Celbio (Milan, Italy); sulforhodamine B (SRB), Thiazolyl Blue Tetrazolium Bromide (MTT) and cisplatin were obtained from Sigma-Aldrich. General chemicals were purchased from Sigma-Aldrich, unless otherwise indicated.

### LogP value determination

The octanol–water partition coefficients for carbene complexes were determined by modification of the reported shake-flask method [42]. Water (50 mL, distilled after milli-Q purification) and n-octanol (50 mL) were shaken together for 72 h to allow saturation of both phases. A solution of the complex was prepared in the water phase (3 × 10^−3^ M) and an equal volume of octanol was added. Biphasic solutions were mixed for ten minutes and then centrifuged for five minutes at 6000 rpm to allow separation. Concentration in both phases was determined by UV-Vis. Reported logP is defined as log[complex]oct/[complex]wat. Final values were reported as mean of three determinations.

### Cell lines and culture conditions

A2780 human ovarian cancer were purchased from the European Collection of Authenticated Cell Cultures (ECACC, a part of Public Health England) (Lot No. 13J012, Sigma-Aldrich). IGROV1 and SKOV3 were a gift of Prof. Joseph R. Bertino (New Brunswick, NJ, USA). Cells were maintained in RPMI1640 medium supplemented with 10% of FCS and antibiotics at 37°C in a 5% CO_2_ atmosphere and sub-cultured twice weekly.

### Antiproliferative activity

Cell proliferation inhibition of Au(NHC) and Au(NHC)_2_ was evaluated against the A2780 cell line according to the method described by Skehan et *al.* [43]. Gold carbene compounds were diluted in DMSO as stock solution (10 mM). Exponentially growing cells were seeded in 96-well microplates in RPMI 1640 supplemented with 10% FCS at a density of 8×10^3^, for 24 h, prior to the addition of two carbene compounds. After 24 h, the medium was removed and replaced with fresh medium containing concentrations of Au(NHC) or Au(NHC)_2_ ranging from 0.003 to 100 μM and incubated for 72 h. For comparison purposes, the cytotoxicity of cisplatin was evaluated under the same experimental conditions. Then, cells were fixed with trichloroacetic acid and stained with sulforhodamine B (SRB) solution (0.4%), rinsed with 1% acetic acid and air-dried. After staining, SRB was dissolved in 10 mM Tris base. Optical density was read in a microplate reader interfaced with the software Microplate Manager/PV version 4.0 (Bio-Rad Laboratories) at 540 nm. The IC_50_ drug concentration resulting in a 50% reduction in the net protein content (as measured by SRB staining) in drug-treated cells as compared with untreated control cells was determined. The effect of carbenes on A2780 cell viability was also assessed with the MTT reduction. Briefly, we performed a time course at 12, 24, 48 and 72 h drug exposure with carbene concentration equal to 72 h-exposure IC_50_ value. At the end of incubation, cells were treated for 1 h at 37°C with 0.5 mg/ml MTT dissolved in PBS. Then, MTT was removed, cells were washed in PBS and 100 μl of stop solution (DMSO) were added. After 15 min of incubation at 37°C, optical density was read in the microplate reader interfaced with the software Microplate Manager/PV version 4.0 (Bio-Rad Laboratories) at 595 nm. Standard MTT proliferation assay was also used to determine IC_50_ values of IGROV1 and SKOV3 cell lines after a 72-hr exposure to Au(NHC) or Au(NHC)_2_ or cisplatin.

### Cell cycle analysis

Cell cycle analysis was assessed as previously reported [67]. Briefly, control, Au(NHC) and Au(NHC)_2_-treated (24, 48 and 72 h) cells were trypsinized, washed with PBS, and fixed in 75% ice-cold ethanol at -20°C overnight. After rehydration with ice-cold PBS, cells were stained with PI/RNase Staining Buffer (BD Biosciences, USA) and analysed using a FACSCanto Flow Cytometer (BD Biosciences). Data were analyzed by ModFit 3.0 software (BD Biosciences).

### Assessment of cell death by flow cytometry

Cell death was analysed by TACS Annexin V/PI Kit (Trevigen) according to the manufacturer's instructions. Briefly, control Au(NHC) and Au(NHC)_2_ treated cells for 72 h were trypsinized, washed with PBS and resuspended in staining solution for 15 min in the dark. Then, cells were immediately analysed by FACSCanto flow cytometer (BD Biosciences). Gated cells were plotted on a dot-plot showing Annexin-V staining and propidium iodide (PI) staining. Index of apoptotic cells was determinate adding Annexin V positive (early apoptotic) cells to Annexin/PI positive ones (late apoptotic).

### Assessment of caspase activity by flow cytometry

Caspase-3, caspase-8 and caspase-9 activity were analysed by flow cytometry as previously reported [68]. Briefly, control Au(NHC) and Au(NHC)_2_ A2780-treated cells for 72 h were trypsinized, washed with PBS and resuspended in FAM-FLICATM Caspases solution (Caspase FLICA kit FAM-DEVD-FMK, ImmunoChemistry Technologies) for 1 h at 37°C, following the manufacturer's instruction. Then, cells were washed twice with PBS, and analysed by FACSCanto flow cytometer (BD Biosciences).

### Two-dimensional gel electrophoresis, image analysis and statistics

Cell growth conditions for proteomic analysis were the same as previously described [23]. Briefly, A2780 cells were treated for 24 h with a concentration of the Au(NHC) or Au(NHC)_2_ equal to 72 h-exposure IC_50_-values and to an equal concentration of DMSO as control. After treatment, cells were washed with phosphate-buffered saline (PBS) and then lysed in RIPA buffer (50 mM Tris-HCl pH 7.0, 1% (v/v) NP-40, 150 mM NaCl, 2 mM ethylene glycol bis(2-aminoethyl ether)tetra-acetic acid, 100 mM NaF) containing a human protease inhibitor cocktail (Sigma-Aldrich). The cells were sonicated (15 s) and protein extracts were clarified by centrifugation at 8000g, 4°C for 15 min. Proteins were precipitated following a chloroform/methanol protocol [69] and the protein pellets were resolved in a buffer containing 8 M urea, 4% (w/v) 3-cholamidopropyl dimethylammonium-1-propane sulfonate (CHAPS), 65 mM dithioerythritol (DTE). The protein concentration was determined by the standard Bradford method (Bio-Rad Laboratories). Isoelectric focusing (IEF) was carried out on an IPGphor system (PROTEAN i12 system Bio-Rad Laboratories) using pH 3–10 gel strips of 18 cm. Strip were actively rehydrated at 30 V for 20 h in 350 μl of sample buffer containing 700 μg of proteins and supplemented with 0.5% (v/v) carrier ampholyte (Bio-Rad Laboratories) and a trace of bromophenol blue. The IEF was performed at 20°C under the following conditions: 250 V for 30 min, 10,000 V for 2 h in gradient, 10,000 V until a total of 43,000 V/h was reached, with a limiting current of the 50 mA per strip. IPG strips were incubated for 15 min in equilibration buffer (50 mM Tris–Cl pH 8.8, 6 M urea, 30% glycerol, 2% SDS) with 6.5 mM DTT and then in equilibration buffer with 2% iodoacetamide for an additional 15 min. The equilibrated strips were placed on top of 9–16% polyacrylamide linear gradient gels (18 cm x 20 cm x1.5 mm) and embedded in 0.5% (w/v) heated low-melting agarose in SDS electrophoresis running buffer (25 m M Tris, 192 m M glycine, 0.1% (w/v) SDS, pH 8.3). The methylene-bisacrilamide was the cross-linker used in the 9–16% gradient. SDS-PAGE was performed in a PROTEAN II xi cell gel electrophoresis unit (Bio-Rad Laboratories) at 10°C and at 40 mA per gel constant current, until the dye front reached the bottom of the gel, according to Hochstrasser et *al.* [70]. Gels were stained with colloidal Coomassie blue silver [71].

To obtain statistically significant results, preparation of cell lysates for proteomic analysis was repeated independently three times for Au(NHC) and Au(NHC)_2_ –treated cells as well as for control cells (biological replicates). To minimize gel-to-gel variation, for each biological replicate, 2-DE were carried out twice (technical replicates). Therefore, for each cell line a total 6 gels were analysed. Colloidal Coomassie blue silver-stained gels were scanned using the Epson expression 1680 PRO scanner. The gel images were saved with a resolution of 300 dpi and in 16-bit TIFF format. Image analysis was carried out using the Progenesis SameSpots software v4.0 (Nonlinear Dynamics, UK), which allows spot detection, background subtraction and protein spot volume quantification. The gel image showing the highest number of spots and the best protein pattern was chosen as the reference image and its spots were then matched across all gels. This reference image was used to quantify and normalize the spot volumes. The spot volumes were normalized in each gel as relative volume (volume percentage), by dividing the raw quantity of each spot by the total quantity of all the spots included in the reference gel. Statistical analysis was performed using default parameters of the Progenesis SameSpots Stat module. The log10-normalized spot volume was used for the analysis as the log transformation improves normality [72]. The univariate data analysis was performed as one-way ANOVA on each spot individually. Then, multivariate statistical analysis was applied on all the ANOVA p-values by the False Discovery Rate (FDR) correction method (*q*-value) [44]. Moreover, we performed a power analysis to assess the number of sample replicates that need to be analysed to confidently discover differentially abundant proteins. The accepted power threshold is >0.8 [45]. We considered statistically differentially abundant spots having a corrected *p*-value (*q*-value) ≤ 0.05 and a power ≥0.8. Moreover, these spots were subjected to multiple comparisons Tukey's post-hoc test using by GraphPad Prism 6.0 software. The statistically differentially abundant spots were analysed by mass spectrometry.

### Mass spectrometry analysis

Electrophoretic spots were manually excised, destained, and acetonitrile (ACN) dehydrated. A trypsin solution (0.25 mg/mL) in 50 mM ammonium bicarbonate was added for in-gel protein digestion by overnight incubation at 37°C. Solutions containing digested peptides were recovered and 20 mL of 1% TFA, 50% ACN were added to each spot and sonicated for 10 min to maximize peptide recovery. At the end, for each spot all recovered peptide solutions were combined and concentrated. From each protein digest, 0.75 mL were spotted onto the MALDI target and allowed to air dry at room temperature. Then, 0.75 mL of matrix solution (saturated solution of α-cyano-4-hydroxycinnamic acid in 50% ACN and 0.5% TFA) was applied to the sample and crystallized by air drying at room temperature for 5 min. Protein identification was carried out by peptide mass fingerprinting (PMF) on a MALDI Ultraflex III TOF/TOF200 mass spectrometer (Bruker Daltonics) equipped with a 200 Hz smartbeamt I laser. MS analysis was performed in the positive reflector mode according to defined parameters, as follows: 80 ns of delay; ion source 1: 25 kV; ion source 2: 21.75 kV; lens voltage: 9.50 kV; reflector voltage: 26.30 kV; and reflector 2 voltage: 14.00 kV. The applied laser wavelength and frequency were 353 nm and 100 Hz, respectively, and the percentage was set to 46%. Final mass spectra were produced by averaging 1500 laser shots targeting five different positions within the spot. Spectra were acquired automatically and the Flex Analysis software version 3.0 (Bruker Daltonics) was used for their analysis and for the assignment of the peaks. The applied software generated a list of peaks up to 200, using a signal-to-noise ratio of 3 as the threshold for peak acceptance. Recorded spectra were calibrated using, as the internal standard, peptides arising from trypsin auto-proteolysis. The mass lists were filtered for contaminant removal: mass matrix related ions, trypsin autolysis and keratin peaks. Protein identification by Peptide Mass Fingerprint (PMF) search was established using MASCOT search engine version 2.1 (Matrix Science, London, UK, http://www.matrixscience.com) through the UniProtKB database (http://www.uniprot.org/). Taxonomy was limited to *Homo sapiens*, a mass tolerance of 150 ppm was allowed, and the number of accepted missed cleavage sites was set to one. Alkylation of cysteine by carbamidomethylation was considered a fixed modification, while oxidation of methionine was considered as a possible modification. The criteria used to accept identifications included the extent of sequence coverage, the number of matched peptides, and a probabilistic score of *p*≤0.05. Peptide digests identified with a Mascot PMF score ≤ 70 were further analysed through MS/MS, using the same mass spectrometer. Two/three PMF peaks showing a high intensity were CID fragmented using Argon as collision gas, and MALDI-TOF/TOF tandem MS was performed in LIFT mode by software-controlled data acquisition. Fragmented ions were analysed using the Flex Analysis software version 3.0 (Bruker Daltonics). The MS/MS database searching was carried out using MASCOT search engine version 2.1 (Matrix Science, London, UK, http://www.matrixscience.com) through the UniProtKB database (http://www.uniprot.org/). The parameters applied for database searching were the same of PMF analysis with a fragment mass tolerance of 0.6 Da. Mascot ion score, peptide coverage by “b” and “y” ions, and expected value were considered for protein identification. Individual ion scores > 25 are significant (*p*≤0.05).

### Bioinformatic functional analysis

To identify statistically over-represented (enriched) Gene Ontology (GO) terms and pathways among the differentially abundant proteins identified by MS analysis, we used the Webgestalt online tool (“WEB-based GEne SeT AnaLysis Toolkit”) (http://bioinfo.vanderbilt.edu/webgestalt/) against Gene Ontology (GO), Kyoto Encyclopedia of Genes and Genomes (KEGG), and Panther databases [46–48]. The list of UniProtKB (http://www.uniprot.org/) accession number (AC) of the identified proteins was loaded into the online tool. After submission of the list, functional classification was performed based on Gene Ontology against the *Homo sapiens* genome. The one-sided Fisher's exact test or the hypergeometric distribution was used to check for significant over-representation. Then, the Benjamini-Hochberg correction was performed to globally correct the p-value controlling the family wide false discovery rate (corrected p-value ≤ 0.1).

### Glucose uptake

Glucose uptake was evaluated in control, Au(NHC) and Au(NHC)_2_ treated cells (24 and 48 h) as previously described [73]. Briefly, controls and treated-cells were incubated for 12 h in starvation medium containing low glucose concentration (DMEM low glucose, supplemented with 10% of FCS) and compounds. Then cells were washed in PBS and incubated with a buffered solution (140 mmol/L NaCl, 20 mmol/L Hepes/Na, 2.5 mmol/L MgSO_4_, 1 mmol/L CaCl_2_, and 5 mmol/L KCl, pH 7.4) containing 0.5 μCi/mL[U-^14^C] glucose. Cells were subsequently washed with cold PBS and lysed with 0.1 mol/L NaOH. Incorporated radioactivity was assayed by liquid scintillation counting and normalized on protein content.

### Measurement of oxygen consumption

Control and Au(NHC) and Au(NHC)_2_ treated cells (24 and 48 h), were trypsinized, washed with PBS and resuspended in complete RPMI medium at the concentration of 10^6^ cells/mL. One ml of cell suspension was transferred to an airtight chamber maintained at 37°C and oxygen consumption was measured using a Clark-type O_2_ electrode (Hansatech) Oxygen content was monitored for at least 10 min. The rate of decrease in oxygen content, related to protein amount was taken as index of the respiratory capability [74].

### Measurement of ATP level

ATP amount was determined with ATP Determination Kit (Molecular Probes) according to manufacturing instruction. ATP amount was normalized on protein content of the same sample.

### Lactate assay

Lactate amount was assayed in cells medium with K-LATE kit (Megazyme) according manufacturer's instructions. Lactate content was normalized on protein content of the same sample.

### Western blot analysis

A2780, IGROV1 and SKOV3 cells were treated for 24, 48 h or 72 h with mono and bis(carbene) at a concentration corresponding to their 72 h-exposure IC_50_-dose. Cells were lysed in RIPA buffer containing a human protease inhibitor cocktail and 20 μg of proteins were separated by 4-20% precast SDS-PAGE (Bio-Rad Laboratories) and transferred to PVDF membranes (Bio-Rad Laboratories). Primary antibody (GAPDH, Sigma Aldrich; AldoA, Thermo Scientific; TPI1, Santa Cruz; COF1, Thermo Scientific; CS, Santa Cruz; Bcl2, and Bax, Santa Cruz) were diluted 1:1000 in 2% Milk and incubated overnight at 4°C. After incubation with horseradish peroxidase (HRP)-conjugated anti-mouse IgG (1:2000) (Santa Cruz Laboratories), immune complexes were detected with the enhanced chemiluminescence (ECL) detection system (GE Healthcare). The PVDF membrane was exposed to autoradiographic films (Pierce) for 1–20 minutes. For quantification, the blot was subjected to densitometric analysis using ImageJ2 program [75]. The intensity of the immunostained bands was normalized with the total protein intensities measured by Coomassie brilliant blue R-250 from the same PVDF membrane blot.

### TrxR inhibition assay

A2780, IGROV1 and SKOV3 cells were treated for 24 h with Au(NHC) or Au(NHC)_2_ concentration corresponding to their 72 h-exposure IC_50_-dose. Cells were lysed in RIPA buffer containing a human protease inhibitor cocktail (Sigma-Aldrich), and 30 μg of proteins were used for the enzymatic assay. The TrxR inhibition was assessed using a commercial colorimetric assay kit (Sigma-Aldrich, CS0170) based on the reduction of 5,5’-dithiobis(2-nitrobenzoic) acid (DTNB) with NADPH to 5-thio-2-nitrobenzoic acid (TNB), according to the manufacturer's instructions. This kit also contains an inhibitor solution of mammalian thioredoxin reductase. Since several enzymes present in biological sample can reduce DTNB, the specific inhibitor is used to determine the reduction of DTNB due only to TrxR activity. Experiments were performed in triplicate. Results were normalized to the cellular protein content.

### ROS formation

ROS generation was measured using 2′,7′-dichlorfluorescein-diacetate (DCF-DA), a fluorogenic dye that binds to ROS. Within the cell, DCFDA is deacetylated by cellular esterase to a non-fluorescent compound, which is later oxidized by cellular ROS into the highly fluorescent compound 2′, 7′-dichlorofluorescein (DCF). A total of 5 μM DCF-DA was added to sub-confluent cells for 3 to 30 min. Cells were lysed in 1 mL RIPA buffer containing 1% Triton X-100 and fluorescence was immediately analysed using a fluorescence spectrophotometer (excitation wavelength: 488 nm, emission wavelength: 510 nm). The fluorescence values were normalized for total protein content. Mitochondrial superoxide anion production was measured using the fluorogenic dye MitoSOX Red (Molecular Probe). Cells suspension was incubated for 15 min with 3 μM MitoSOX Red, washed in PBS and analysed by FACSCanto flow cytometer (BD Biosciences).

### Measurement of mitochondrial potential membrane

Mitochondrial membrane potential was assessed with 1,1′,3,3,3′,3′-hexamethylindodicarbo- cyanine iodide, DiIC1(5) (1,1’,3,3,3,3’-hexamethylindodicarbo-cyanine iodide) (MitoProbe^TM^), a cationic cyanine dyes that accumulates primarily in mitochondria with active membrane potentials. Cells suspensions were washed twice with PBS and incubated, in the dark, for 20 min. at 37°C with DiIC1(5) (50 nM) in PBS. After labelling, cells were washed, resuspended in PBS and analysed by FACSCanto flow cytometer (BD Biosciences).

### Statistical analysis

The non-proteomic experiments were carried out, at least, in triplicate. Statistical analysis was performed by one-way ANOVA test followed by Tuckey's multiple comparisons test using Graphpad Prism 6. A *p*-value≤0.05 was considered statistically significant. Results were reported as mean ±SD.

This study was supported by contributions of Associazione Italiana per la Ricerca sul Cancro, Milan (AIRC-IG16049) and Istituto Toscano Tumori (Messori-ITT-2015), Beneficentia Stiftung and Ente Cassa di Risparmio di Firenze, to LMe; by AIRC (IG 15565) to EM; and by Ente Cassa di Risparmio di Firenze, (no. 2014/0706 to EM, no. 2014/0969 to SN).

## SUPPLEMENTARY MATERIALS FIGURES AND TABLES




