# Supplementary material for: Antiproliferative effects of two gold(I)-N-heterocyclic carbene complexes in A2780 human ovarian cancer cells: a comparative proteomic study
Source: Oncotarget. 2018 Jun 15;9(46):28042–68. doi: 10.18632/oncotarget.25556 (PMC6021324; doi:10.18632/oncotarget.25556)
Supplement: Supplementary file 1 [file oncotarget-09-28042-s001.pdf]

Antiproliferative effects of two gold(I)-N-heterocyclic carbene complexes in A2780 human ovarian cancer cells: a comparative proteomic study

SUPPLEMENTARY MATERIALS

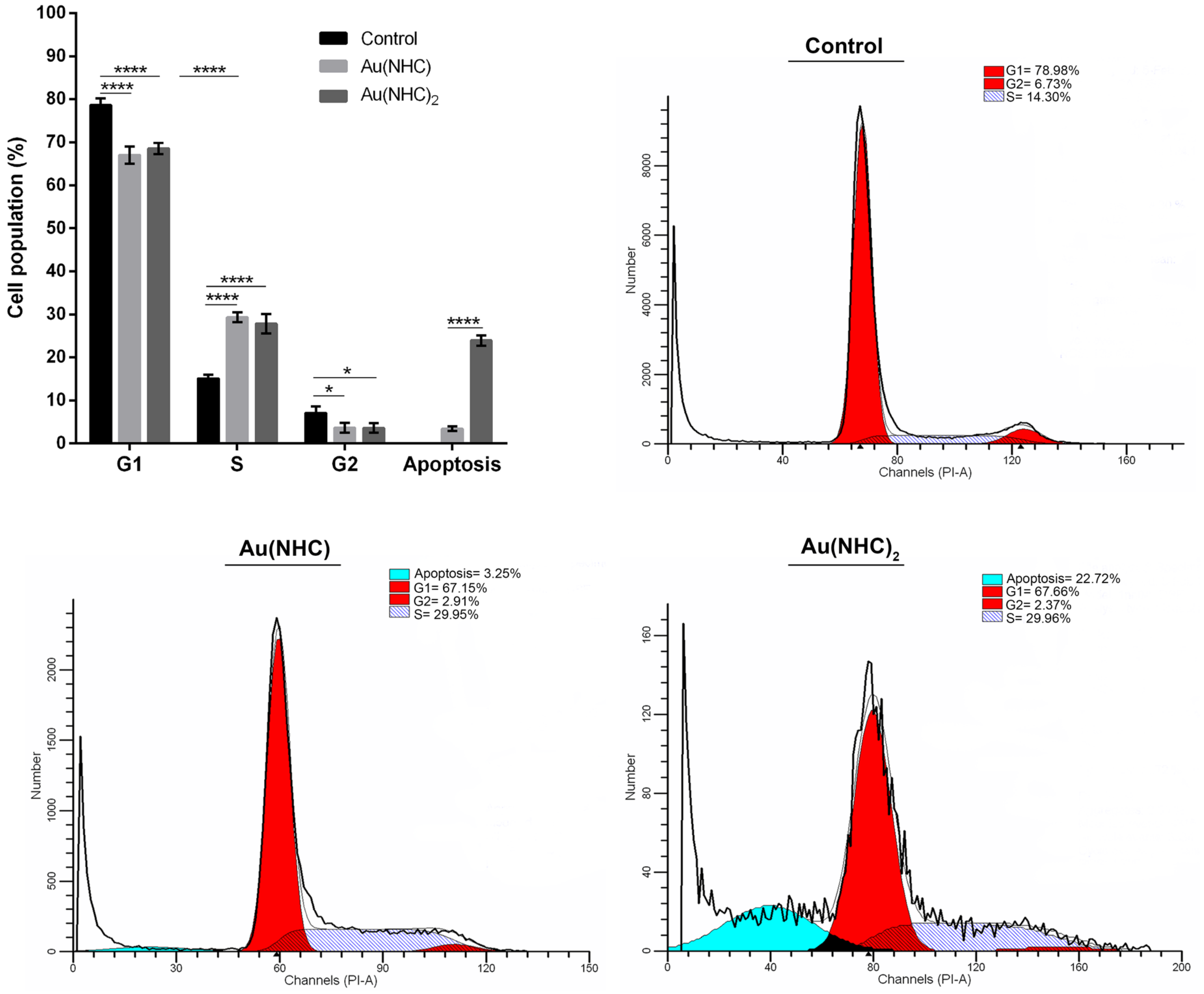

Supplementary Figure 1: Cell cycle distribution after 72 h of gold(I)-N-heterocyclic carbene treatment. Cell cycle distribution of A2780 cell line was assessed by flow cytometric analysis. Histogram reports the cell percentage in G1, S and G2 phase of cell cycle after 72 h of treatment with the 72 h-exposure IC_{50}-dose of Au(NHC) and $Au(NHC)_2$. Flow cytometric images are representative of three independent experiments. Results are reported as mean ± SD. The statistical analysis was carried out using one-way ANOVA test followed by Tuckey's multiple comparisons test using Graphpad Prism v 6.0 ($^{*}p<0.05$, $^{****}p<0.0001$).

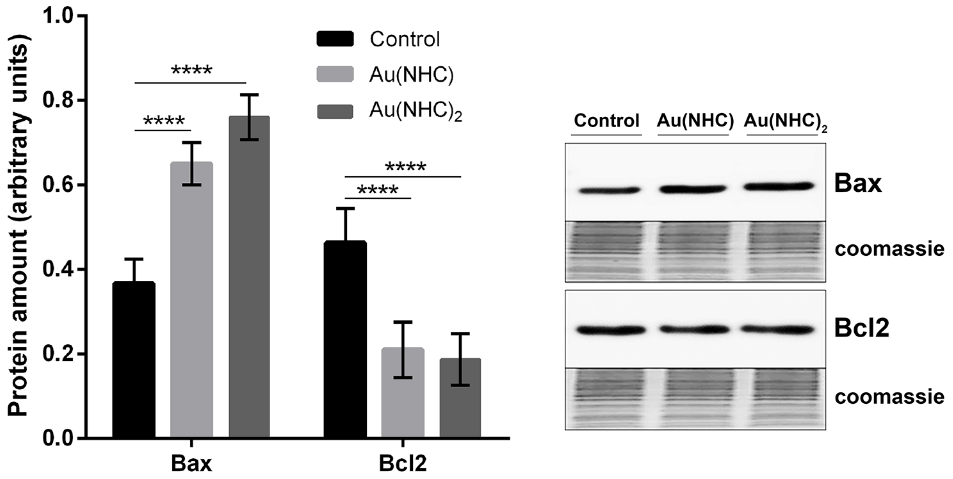

Supplementary Figure 2: Apoptosis induction by Au(NHC) and Au(NHC)[2] in A2780 cell line. A2780 cells were treated with Au(NHC) and Au(NHC)$_2$ for 72 h with their 72 h-exposure IC$_{50}$-dose and apoptosis induction was confirmed by western blot analysis of pro-apoptotic Bax and anti-apoptotic Bcl2 level. Representative Immunoblots are shown together with the corresponding Coomassie-stained PVDF membranes. All the experiments were performed in triplicate. Histogram reports normalized mean relative-integrated-density ± SD values of the Bax and Bcl2 bands. The statistical analysis was carried out using one-way ANOVA test followed by Tuckey's multiple comparisons test using Graphpad Prism v 6.0 ($^{****}p<0.0001$). In detail, western blot analysis pointed out an increment in Bax level of about 2.7-fold upon Au(NHC) exposure and, of about 2.8-fold upon Au(NHC)$_2$ exposure. Conversely, the Bcl2 level was decreased of about 2-fold in monocarbene-treated cells and of about 2.5-fold in dicarbene treated cells.

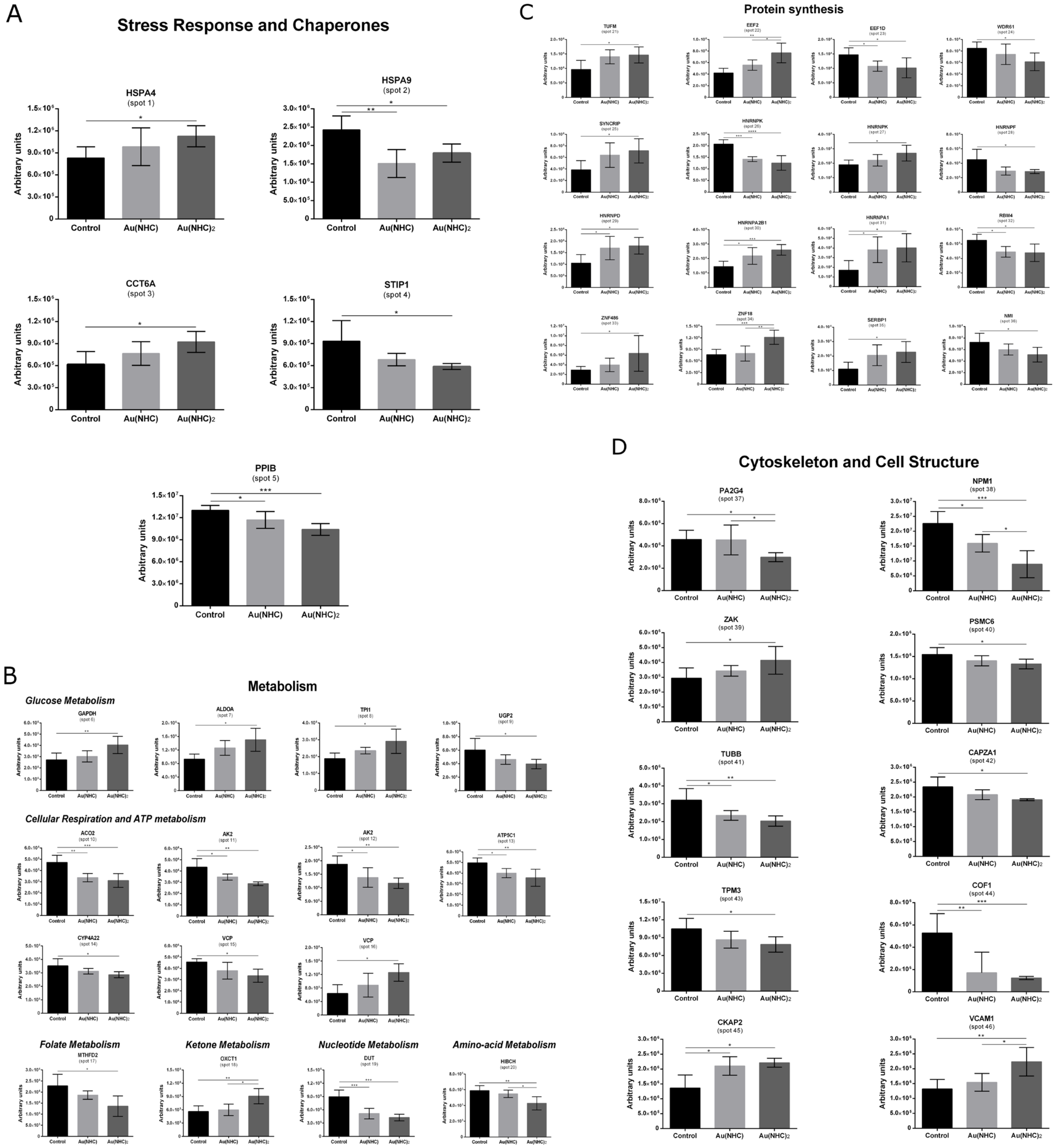

Supplementary Figure 3: Quantitative analysis of the 51 differentially expressed protein spots identified by mass spectrometry. **(A)** Stress Response and Chaperones; **(B)** Metabolism; **(C)** Protein synthesis; **(D)** Cytoskeleton and Cell Structure; **(E)** Cell redox homeostasis, Transport, DNA replication and repair. Histograms represent the abundance of each spot (normalized volume, arbitrary units) in A2780 control, Au(NHC)- and $Au(NHC)_2$-treated cells. ANOVA test was performed by Progenesis SameSpots 4.0 software (Nonlinear Dynamic, UK) to determine if the relative change was statistically significant (p-value<0.05). All spots show a q-value (False Discovery Rate) <0.1. The significant differences between groups were calculated with GraphPad Prism6.0 software, using Tukey correction for multiple comparison (see Table 2). ($^*p<0.05$), ($^{**}p<0.01$), ($^{***}p<0.001$), ($^{****}p<0.0001$). For details, see Materials and Methods.

Supplementary Table 1: MS/MS analysis of differentially expressed protein spots identified by MALDI-TOF/TOF MS with a PMF score ≤ 70

Spot No[a]	Protein name	Gene Name	AC[b]	Observed Mr(kDa)/ pI[c]	Theoretical Mr (kDa)/ pI	Mascot search results PMF score[d]	Matched Pept.[e0]	Seq. Coverage (%)[f]	MS/MS score[g]	Peptide Identified
**Stress Response and Chaperones**										
1	Heat shock 70 kDa protein 4	HSPA4	P34932	94.2/5.1	95.1/5.1	62	7/15	11	50<br>42	[391 – 405] R.EFSITDVVPYPISLR.W<br>[574 – 591] K.TSTVDLPIENQLLWQIDR
2	Stress-70 protein, mitochondrial	HSPA9	P38646	63.0/5.2	73.9/5.9	62	6/13	12	70	[499 – 513] K.LLGQFTLIGIPPAPR.G
Metabolism										
7	Fructose-bisphosphate aldolase A	ALDOA	P04075	35.1/7.5	39.8/8.3	69	8/26	29	57<br>46	[88 – 99] K.ADDGRPFPQVIK.S<br>[244 – 258] K.FSHEEIAMATVTALR.R
11	Adenylate kinase 2, mitochondrial	AK2	P54819	28.9/7.5	26.7/7.7	62	6/24	25	62	[94 – 103] K.NGFLLDGFPR.T
12	Adenylate kinase 2, mitochondrial	AK2	P54819	28.9/7.6	26.7/7.7	62	6/26	25	51	[187 – 202] R.LQAYHTQTTPLIEYYR.K
13	ATP synthase subunit gamma, mitochondrial	ATP5C1	P36542	26.3/7.5	33.0/9.2	62	6/28	15	74	[127 – 138] K.EVMLVGIGDKIR.G
14	Cytochrome P450 4A22	CYP4A22	Q5TCH4	39.2/6.8	59.7/9.2	59	7p/22	15	82<br>75	[34 – 46] K.AAQLYLHRQWLLK.A<br>[378 – 391] K.EALRLYPPVPGIGR.E
Protein synthesis										
23	Elongation factor 1-delta	EEF1D	P29692	39.2/4.7	31.2/4.9	58	6/32	30	66	[60 – 83] K.SLAGSSGPGASSGTSGDHGELVVR.I
30	Heterogeneous nuclear ribonucleoproteins A2/B1	HNRNPA2B1	P22626	32.2/7.4	37.5/8.9	66	7/27	18	58<br>61	[23 – 38] K.LFIGGLSFETTEESLR.N<br>[130 – 137] R.DYFEEYGK.I
33	Zinc finger protein 486	ZNF486	Q96H40	54.1/8.1	55.1/9.3	66	8/28	22	49<br>56	[7 – 16] R.SLEMESLQFR.D<br>[138 – 152] R.GYNGLNQCLTTTQSK.I
34	Zinc finger protein 18	ZNF18	P17022	48.6/5.3	62.3/5.7	66	8p/57	20	70	[281 – 290] K.IPRPTCIGDR.Q
35	Plasminogen activator inhibitor 1 RNA-binding protein	SERBP1	Q8NC51	54.1/8.6	45.0/8.7	66	9/26	25	88	[112 – 122] R.RPDQQLQGEGK.I
Cytoskeleton and Cell Structure										
39	Mitogen-activated protein kinase kinase kinase MLT (fragment)	ZAK	Q9NYL2	23.1/7.3	91.2/7.9	60	9/41	17	65	[355 – 369] K.GDSSAEMSVYASLFK.E
43	Tropomyosin alpha-3 chain	TPM3	P06753	33.2/4.6	32.9/4.7	70	6/16	14	100<br>92	[93 – 102] R.IQLVEEELDR.A<br>[170 – 179] K.LVIIEGDLER.T
46	Vascular cell adhesion protein 1 (fragment)	VCAM1	P19320	31.9/5.2	82.3/5.1	63	6/15	16	75	[443 – 459] K.GETILENIEFLEDTDMK.S
Transport										
49	Oxysterol-binding protein-related protein 1 (fragment)	OSBPL1A	Q9BXW6	46.1/4.7	109.8/5.9	57	7/13	8	59	[519 – 531] K.DCGGGDALSNGIK.K
DNA replication and repair										
51	Single-stranded DNA-binding protein, mitochondrial	SSBP	Q04837	15.2/8.2	17.2/9.6	70	6/23	37	75<br>91	[52 – 66] K.NPVTIFSLATNEMWR.S<br>[87 – 95] R.ISVFRPGLR.D

The complete list of the identified protein spots is reported in Table 2.

a Spot numbers match those reported in the representative 2-DE images shown in Figure 4A.

b Accession number in Swiss-Prot/UniProtKB (http://www.uniprot.org/).

c Observed Mr/pI, based on the calculation using Progenesis SameSpots 4.0 software

d MASCOT PMF (Peptide Mass Fingerprint) score (Matrix Science, London, UK; http://www.matrixscience.com). MS matching score greater than 56 was required for a significant MS hit (*p*-value<0.05).

e Number of matched peptides correspond to peptide masses matching the top hit from Ms-Fit PMF, searched peptides are also reported.

f Sequence coverage = (number of the identified residues/total number of amino acid residues in the protein sequence) x100%.

g MASCOT MS/MS score (Matrix Science, London, UK; http://www.matrixscience.com). Ion score greater than 25 was significant (*p*-value<0.05).

Supplementary Table 2: Overrepresentation Enrichment Analysis (ORA) of GO terms and pathways obtained from the identified protein list, using Webgestalt functional enrichment analysis web tool (http://www.webgestalt.org/option.php).

See Supplementary File 1
