# Supplementary material for: Antiproliferative effects of two gold(I)-N-heterocyclic carbene complexes in A2780 human ovarian cancer cells: a comparative proteomic study
Source: Oncotarget. 2018 Jun 15;9(46):28042–68. doi: 10.18632/oncotarget.25556 (PMC6021324; doi:10.18632/oncotarget.25556)
Supplement: Supplementary file 2 [file oncotarget-09-28042-s002.docx]

**Supplementary Table 2:** Overrepresentation Enrichment Analysis (ORA) of GO terms and pathways obtained from the identified protein list, using Webgestalt functional enrichment analysis web tool (http://www.webgestalt.org/option.php).

| ***GO Biological Process*** | | | |
| --- | --- | --- | --- |
| **ID:GO:0010608      Name: posttranscriptional regulation of gene expression** | | | |
| C=454; O=10; E=1.35; R=7.4; PValue=6.29e-07; FDR=4.79e-04 | | | |
| **AC** | **Gene Symbol** | **Gene Name** | **Entrez Gene** |
| O60506 | SYNCRIP | synaptotagmin binding cytoplasmic RNA interacting protein | 10492 |
| P13639 | EEF2 | eukaryotic translation elongation factor 2 | 1938 |
| P04406 | GAPDH | glyceraldehyde-3-phosphate dehydrogenase | [2597](http://www.ncbi.nlm.nih.gov/sites/entrez?db=gene&cmd=Retrieve&dopt=Graphics&list_uids=2597) |
| Q8NC51 | SERBP1 | SERPINE1 mRNA binding protein 1 | [26135](http://www.ncbi.nlm.nih.gov/sites/entrez?db=gene&cmd=Retrieve&dopt=Graphics&list_uids=26135) |
| P22626 | HNRNPA2B1 | heterogeneous nuclear ribonucleoprotein A2/B1 | [3181](http://www.ncbi.nlm.nih.gov/sites/entrez?db=gene&cmd=Retrieve&dopt=Graphics&list_uids=3181) |
| Q14103 | HNRNPD | heterogeneous nuclear ribonucleoprotein D | [3184](http://www.ncbi.nlm.nih.gov/sites/entrez?db=gene&cmd=Retrieve&dopt=Graphics&list_uids=3184) |
| P06748 | NPM1 | nucleophosmin | [4869](http://www.ncbi.nlm.nih.gov/sites/entrez?db=gene&cmd=Retrieve&dopt=Graphics&list_uids=4869) |
| Q9UQ80 | PA2G4 | proliferation-associated 2G4 | [5036](http://www.ncbi.nlm.nih.gov/sites/entrez?db=gene&cmd=Retrieve&dopt=Graphics&list_uids=5036) |
| P62333 | PSMC6 | proteasome 26S subunit, ATPase 6 | [5706](http://www.ncbi.nlm.nih.gov/sites/entrez?db=gene&cmd=Retrieve&dopt=Graphics&list_uids=5706) |
| Q9BWF3 | RBM4 | RNA binding motif protein 4 | [5936](http://www.ncbi.nlm.nih.gov/sites/entrez?db=gene&cmd=Retrieve&dopt=Graphics&list_uids=5936) |

| **ID:GO:0034248      Name: regulation of cellular amide metabolic process** | | | |
| --- | --- | --- | --- |
| C=359; O=8; E=1.07; R=7.49; PValue=9e-06; FDR=3.43e-03 | | | |
| **AC** | **Gene Symbol** | **Gene Name** | **Entrez Gene** |
| O60506 | SYNCRIP | synaptotagmin binding cytoplasmic RNA interacting protein | [10492](http://www.ncbi.nlm.nih.gov/sites/entrez?db=gene&cmd=Retrieve&dopt=Graphics&list_uids=10492) |
| P13639 | EEF2 | eukaryotic translation elongation factor 2 | [1938](http://www.ncbi.nlm.nih.gov/sites/entrez?db=gene&cmd=Retrieve&dopt=Graphics&list_uids=1938) |
| P04406 | GAPDH | glyceraldehyde-3-phosphate dehydrogenase | [2597](http://www.ncbi.nlm.nih.gov/sites/entrez?db=gene&cmd=Retrieve&dopt=Graphics&list_uids=2597) |
| P22626 | HNRNPA2B1 | heterogeneous nuclear ribonucleoprotein A2/B1 | [3181](http://www.ncbi.nlm.nih.gov/sites/entrez?db=gene&cmd=Retrieve&dopt=Graphics&list_uids=3181) |
| Q14103 | HNRNPD | heterogeneous nuclear ribonucleoprotein D | [3184](http://www.ncbi.nlm.nih.gov/sites/entrez?db=gene&cmd=Retrieve&dopt=Graphics&list_uids=3184) |
| P06748 | NPM1 | nucleophosmin | [4869](http://www.ncbi.nlm.nih.gov/sites/entrez?db=gene&cmd=Retrieve&dopt=Graphics&list_uids=4869) |
| Q9UQ80 | PA2G4 | proliferation-associated 2G4 | [5036](http://www.ncbi.nlm.nih.gov/sites/entrez?db=gene&cmd=Retrieve&dopt=Graphics&list_uids=5036) |
| Q9BWF3 | RBM4 | RNA binding motif protein 4 | [5936](http://www.ncbi.nlm.nih.gov/sites/entrez?db=gene&cmd=Retrieve&dopt=Graphics&list_uids=5936) |

| **ID:GO:0071897      Name: DNA biosynthetic process** | | | |
| --- | --- | --- | --- |
| C=194; O=6; E=0.58; R=10.39; PValue=2.17e-05; FDR=5.52e-03 | | | |
| **AC** | **Gene Symbol** | **Gene Name** | **Entrez Gene** |
| P09651 | HNRNPA1 | heterogeneous nuclear ribonucleoprotein A1 | [3178](http://www.ncbi.nlm.nih.gov/sites/entrez?db=gene&cmd=Retrieve&dopt=Graphics&list_uids=3178) |
| P22626 | HNRNPA2B1 | heterogeneous nuclear ribonucleoprotein A2/B1 | [3181](http://www.ncbi.nlm.nih.gov/sites/entrez?db=gene&cmd=Retrieve&dopt=Graphics&list_uids=3181) |
| Q14103 | HNRNPD | heterogeneous nuclear ribonucleoprotein D | [3184](http://www.ncbi.nlm.nih.gov/sites/entrez?db=gene&cmd=Retrieve&dopt=Graphics&list_uids=3184) |
| P62937 | PPIA | peptidylprolyl isomerase A | [5478](http://www.ncbi.nlm.nih.gov/sites/entrez?db=gene&cmd=Retrieve&dopt=Graphics&list_uids=5478) |
| P55072 | VCP | valosin containing protein | [7415](http://www.ncbi.nlm.nih.gov/sites/entrez?db=gene&cmd=Retrieve&dopt=Graphics&list_uids=7415) |
| P40227 | CCT6A | chaperonin containing TCP1 subunit 6A | [908](http://www.ncbi.nlm.nih.gov/sites/entrez?db=gene&cmd=Retrieve&dopt=Graphics&list_uids=908) |

| **ID:GO:0009123      Name: nucleoside monophosphate metabolic process** | | | |
| --- | --- | --- | --- |
| C=308; O=7; E=0.92; R=7.64; PValue=3.09e-05; FDR=5.88e-03 | | | |
| **AC** | **Gene Symbol** | **Gene Name** | **Entrez Gene** |
| P33316 | DUT | deoxyuridine triphosphatase | [1854](http://www.ncbi.nlm.nih.gov/sites/entrez?db=gene&cmd=Retrieve&dopt=Graphics&list_uids=1854) |
| P54819 | AK2 | adenylate kinase 2 | [204](http://www.ncbi.nlm.nih.gov/sites/entrez?db=gene&cmd=Retrieve&dopt=Graphics&list_uids=204) |
| P04075 | ALDOA | aldolase, fructose-bisphosphate A | [226](http://www.ncbi.nlm.nih.gov/sites/entrez?db=gene&cmd=Retrieve&dopt=Graphics&list_uids=226) |
| P04406 | GAPDH | glyceraldehyde-3-phosphate dehydrogenase | [2597](http://www.ncbi.nlm.nih.gov/sites/entrez?db=gene&cmd=Retrieve&dopt=Graphics&list_uids=2597) |
| P36542 | ATP5C1 | ATP synthase, H+ transporting, mitochondrial F1 complex, gamma polypeptide 1 | [509](http://www.ncbi.nlm.nih.gov/sites/entrez?db=gene&cmd=Retrieve&dopt=Graphics&list_uids=509) |
| P60174 | TPI1 | triosephosphate isomerase 1 | [7167](http://www.ncbi.nlm.nih.gov/sites/entrez?db=gene&cmd=Retrieve&dopt=Graphics&list_uids=7167) |
| P55072 | VCP | valosin containing protein | [7415](http://www.ncbi.nlm.nih.gov/sites/entrez?db=gene&cmd=Retrieve&dopt=Graphics&list_uids=7415) |

| [**ID:GO:0006091      Name: generation of precursor metabolites and energy**](http://amigo.geneontology.org/amigo/term/GO:0006091) | | | |
| --- | --- | --- | --- |
| C=365; O=7; E=1.09; R=6.44; PValue=9.04e-05; FDR=1.38e-02 | | | |
| **AC** | **Gene Symbol** | **Gene Name** | **Entrez Gene** |
| P04075 | ALDOA | aldolase, fructose-bisphosphate A | [226](http://www.ncbi.nlm.nih.gov/sites/entrez?db=gene&cmd=Retrieve&dopt=Graphics&list_uids=226) |
| P04406 | GAPDH | glyceraldehyde-3-phosphate dehydrogenase | [2597](http://www.ncbi.nlm.nih.gov/sites/entrez?db=gene&cmd=Retrieve&dopt=Graphics&list_uids=2597) |
| Q99798 | ACO2 | aconitase 2 | [50](http://www.ncbi.nlm.nih.gov/sites/entrez?db=gene&cmd=Retrieve&dopt=Graphics&list_uids=50) |
| P36542 | ATP5C1 | ATP synthase, H+ transporting, mitochondrial F1 complex, gamma polypeptide 1 | [509](http://www.ncbi.nlm.nih.gov/sites/entrez?db=gene&cmd=Retrieve&dopt=Graphics&list_uids=509) |
| P60174 | TPI1 | triosephosphate isomerase 1 | [7167](http://www.ncbi.nlm.nih.gov/sites/entrez?db=gene&cmd=Retrieve&dopt=Graphics&list_uids=7167) |
| Q16851 | UGP2 | UDP-glucose pyrophosphorylase 2 | [7360](http://www.ncbi.nlm.nih.gov/sites/entrez?db=gene&cmd=Retrieve&dopt=Graphics&list_uids=7360) |
| P55072 | VCP | valosin containing protein | [7415](http://www.ncbi.nlm.nih.gov/sites/entrez?db=gene&cmd=Retrieve&dopt=Graphics&list_uids=7415) |

| **ID:GO:0046939      Name: nucleotide phosphorylation** | | | |
| --- | --- | --- | --- |
| C=90; O=4; E=0.27; R=14.93; PValue=1.44e-04; FDR=1.83e-02 | | | |
| **AC** | **Gene Symbol** | **Gene Name** | **Entrez Gene** |
| P54819 | AK2 | adenylate kinase 2 | [204](http://www.ncbi.nlm.nih.gov/sites/entrez?db=gene&cmd=Retrieve&dopt=Graphics&list_uids=204) |
| P04075 | ALDOA | aldolase, fructose-bisphosphate A | [226](http://www.ncbi.nlm.nih.gov/sites/entrez?db=gene&cmd=Retrieve&dopt=Graphics&list_uids=226) |
| P04406 | GAPDH | glyceraldehyde-3-phosphate dehydrogenase | [2597](http://www.ncbi.nlm.nih.gov/sites/entrez?db=gene&cmd=Retrieve&dopt=Graphics&list_uids=2597) |
| P60174 | TPI1 | triosephosphate isomerase 1 | [7167](http://www.ncbi.nlm.nih.gov/sites/entrez?db=gene&cmd=Retrieve&dopt=Graphics&list_uids=7167) |

| **ID:GO:0009141      Name: nucleoside triphosphate metabolic process** | | | |
| --- | --- | --- | --- |
| C=290; O=6; E=0.86; R=6.95; PValue=2.01e-04; FDR=2.08e-02 | | | |
| **AC** | **Gene Symbol** | **Gene Name** | **Entrez Gene** |
| P33316 | DUT | deoxyuridine triphosphatase | [1854](http://www.ncbi.nlm.nih.gov/sites/entrez?db=gene&cmd=Retrieve&dopt=Graphics&list_uids=1854) |
| P04075 | ALDOA | aldolase, fructose-bisphosphate A | [226](http://www.ncbi.nlm.nih.gov/sites/entrez?db=gene&cmd=Retrieve&dopt=Graphics&list_uids=226) |
| P04406 | GAPDH | glyceraldehyde-3-phosphate dehydrogenase | [2597](http://www.ncbi.nlm.nih.gov/sites/entrez?db=gene&cmd=Retrieve&dopt=Graphics&list_uids=2597) |
| P36542 | ATP5C1 | ATP synthase, H+ transporting, mitochondrial F1 complex, gamma polypeptide 1 | [509](http://www.ncbi.nlm.nih.gov/sites/entrez?db=gene&cmd=Retrieve&dopt=Graphics&list_uids=509) |
| P60174 | TPI1 | triosephosphate isomerase 1 | [7167](http://www.ncbi.nlm.nih.gov/sites/entrez?db=gene&cmd=Retrieve&dopt=Graphics&list_uids=7167) |
| P55072 | VCP | valosin containing protein | [7415](http://www.ncbi.nlm.nih.gov/sites/entrez?db=gene&cmd=Retrieve&dopt=Graphics&list_uids=7415) |

| **ID:GO:1901657      Name: glycosyl compound metabolic process** | | | |
| --- | --- | --- | --- |
| C=421; O=7; E=1.25; R=5.59; PValue=2.19e-04; FDR=2.08e-02 | | | |
| **AC** | **Gene Symbol** | **Gene Name** | **Entrez Gene** |
| P33316 | DUT | deoxyuridine triphosphatase | [1854](http://www.ncbi.nlm.nih.gov/sites/entrez?db=gene&cmd=Retrieve&dopt=Graphics&list_uids=1854) |
| P54819 | AK2 | adenylate kinase 2 | [204](http://www.ncbi.nlm.nih.gov/sites/entrez?db=gene&cmd=Retrieve&dopt=Graphics&list_uids=204) |
| P04075 | ALDOA | aldolase, fructose-bisphosphate A | [226](http://www.ncbi.nlm.nih.gov/sites/entrez?db=gene&cmd=Retrieve&dopt=Graphics&list_uids=226) |
| P04406 | GAPDH | glyceraldehyde-3-phosphate dehydrogenase | [2597](http://www.ncbi.nlm.nih.gov/sites/entrez?db=gene&cmd=Retrieve&dopt=Graphics&list_uids=2597) |
| P36542 | ATP5C1 | ATP synthase, H+ transporting, mitochondrial F1 complex, gamma polypeptide 1 | [509](http://www.ncbi.nlm.nih.gov/sites/entrez?db=gene&cmd=Retrieve&dopt=Graphics&list_uids=509) |
| P60174 | TPI1 | triosephosphate isomerase 1 | [7167](http://www.ncbi.nlm.nih.gov/sites/entrez?db=gene&cmd=Retrieve&dopt=Graphics&list_uids=7167) |
| P55072 | VCP | valosin containing protein | [7415](http://www.ncbi.nlm.nih.gov/sites/entrez?db=gene&cmd=Retrieve&dopt=Graphics&list_uids=7415) |

| **ID:GO:0009132      Name: nucleoside diphosphate metabolic process** | | | |
| --- | --- | --- | --- |
| C=109; O=4; E=0.32; R=12.33; PValue=3.01e-04; FDR=2.55e-02 | | | |
| **AC** | **Gene Symbol** | **Gene Name** | **Entrez Gene** |
| P54819 | AK2 | adenylate kinase 2 | [204](http://www.ncbi.nlm.nih.gov/sites/entrez?db=gene&cmd=Retrieve&dopt=Graphics&list_uids=204) |
| P04075 | ALDOA | aldolase, fructose-bisphosphate A | [226](http://www.ncbi.nlm.nih.gov/sites/entrez?db=gene&cmd=Retrieve&dopt=Graphics&list_uids=226) |
| P04406 | GAPDH | glyceraldehyde-3-phosphate dehydrogenase | [2597](http://www.ncbi.nlm.nih.gov/sites/entrez?db=gene&cmd=Retrieve&dopt=Graphics&list_uids=2597) |
| P60174 | TPI1 | triosephosphate isomerase 1 | [7167](http://www.ncbi.nlm.nih.gov/sites/entrez?db=gene&cmd=Retrieve&dopt=Graphics&list_uids=7167) |

| **ID:GO:0060249      Name: anatomical structure homeostasis** | | | |
| --- | --- | --- | --- |
| C=341; O=6; E=1.02; R=5.91; PValue=4.8e-04; FDR=3.66e-02 | | | |
| **AC** | **Gene Symbol** | **Gene Name** | **Entrez Gene** |
| P04075 | ALDOA | aldolase, fructose-bisphosphate A | [226](http://www.ncbi.nlm.nih.gov/sites/entrez?db=gene&cmd=Retrieve&dopt=Graphics&list_uids=226) |
| P09651 | HNRNPA1 | heterogeneous nuclear ribonucleoprotein A1 | [3178](http://www.ncbi.nlm.nih.gov/sites/entrez?db=gene&cmd=Retrieve&dopt=Graphics&list_uids=3178) |
| P22626 | HNRNPA2B1 | heterogeneous nuclear ribonucleoprotein A2/B1 | [3181](http://www.ncbi.nlm.nih.gov/sites/entrez?db=gene&cmd=Retrieve&dopt=Graphics&list_uids=3181) |
| Q14103 | HNRNPD | heterogeneous nuclear ribonucleoprotein D | [3184](http://www.ncbi.nlm.nih.gov/sites/entrez?db=gene&cmd=Retrieve&dopt=Graphics&list_uids=3184) |
| Q06830 | PRDX1 | peroxiredoxin 1 | [5052](http://www.ncbi.nlm.nih.gov/sites/entrez?db=gene&cmd=Retrieve&dopt=Graphics&list_uids=5052) |
| P40227 | CCT6A | chaperonin containing TCP1 subunit 6A | [908](http://www.ncbi.nlm.nih.gov/sites/entrez?db=gene&cmd=Retrieve&dopt=Graphics&list_uids=908) |

| ***GO Molecular Function*** | | | |
| --- | --- | --- | --- |
| [**ID:GO:0098631      Name:protein binding involved in cell adhesion**](http://amigo.geneontology.org/amigo/term/GO:0098631) | | | |
| C=293; O=7; E=1; R=7; PValue=5.27e-05; FDR=1.26e-02 | | | |
| **AC** | **Gene Symbol** | **Gene Name** | **Entrez Gene** |
| P29692 | EEF1D | eukaryotic translation elongation factor 1 delta | [1936](http://www.ncbi.nlm.nih.gov/sites/entrez?db=gene&cmd=Retrieve&dopt=Graphics&list_uids=1936) |
| P13639 | EEF2 | eukaryotic translation elongation factor 2 | [1938](http://www.ncbi.nlm.nih.gov/sites/entrez?db=gene&cmd=Retrieve&dopt=Graphics&list_uids=1938) |
| P04075 | ALDOA | aldolase, fructose-bisphosphate A | [226](http://www.ncbi.nlm.nih.gov/sites/entrez?db=gene&cmd=Retrieve&dopt=Graphics&list_uids=226) |
| Q8NC51 | SERBP1 | SERPINE1 mRNA binding protein 1 | [26135](http://www.ncbi.nlm.nih.gov/sites/entrez?db=gene&cmd=Retrieve&dopt=Graphics&list_uids=26135) |
| Q06830 | PRDX1 | peroxiredoxin 1 | [5052](http://www.ncbi.nlm.nih.gov/sites/entrez?db=gene&cmd=Retrieve&dopt=Graphics&list_uids=5052) |
| P52907 | CAPZA1 | capping actin protein of muscle Z-line alpha subunit 1 | [829](http://www.ncbi.nlm.nih.gov/sites/entrez?db=gene&cmd=Retrieve&dopt=Graphics&list_uids=829) |
| Q9Y265 | RUVBL1 | RuvB like AAA ATPase 1 | [8607](http://www.ncbi.nlm.nih.gov/sites/entrez?db=gene&cmd=Retrieve&dopt=Graphics&list_uids=8607) |

| **ID:GO:0050839      Name: cell adhesion molecule binding** | | | |
| --- | --- | --- | --- |
| C=445; O=8; E=1.52; R=5.27; PValue=1.08e-04; FDR=1.26e-02 | | | |
| **AC** | **Gene Symbol** | **Gene Name** | **Entrez Gene** |
| P29692 | EEF1D | eukaryotic translation elongation factor 1 delta | [1936](http://www.ncbi.nlm.nih.gov/sites/entrez?db=gene&cmd=Retrieve&dopt=Graphics&list_uids=1936) |
| P13639 | EEF2 | eukaryotic translation elongation factor 2 | [1938](http://www.ncbi.nlm.nih.gov/sites/entrez?db=gene&cmd=Retrieve&dopt=Graphics&list_uids=1938) |
| P04075 | ALDOA | aldolase, fructose-bisphosphate A | [226](http://www.ncbi.nlm.nih.gov/sites/entrez?db=gene&cmd=Retrieve&dopt=Graphics&list_uids=226) |
| Q8NC51 | SERBP1 | SERPINE1 mRNA binding protein 1 | [26135](http://www.ncbi.nlm.nih.gov/sites/entrez?db=gene&cmd=Retrieve&dopt=Graphics&list_uids=26135) |
| Q06830 | PRDX1 | peroxiredoxin 1 | [5052](http://www.ncbi.nlm.nih.gov/sites/entrez?db=gene&cmd=Retrieve&dopt=Graphics&list_uids=5052) |
| P19320 | VCAM1 | vascular cell adhesion molecule 1 | [7412](http://www.ncbi.nlm.nih.gov/sites/entrez?db=gene&cmd=Retrieve&dopt=Graphics&list_uids=7412) |
| P52907 | CAPZA1 | capping actin protein of muscle Z-line alpha subunit 1 | [829](http://www.ncbi.nlm.nih.gov/sites/entrez?db=gene&cmd=Retrieve&dopt=Graphics&list_uids=829) |
| Q9Y265 | RUVBL1 | RuvB like AAA ATPase 1 | [8607](http://www.ncbi.nlm.nih.gov/sites/entrez?db=gene&cmd=Retrieve&dopt=Graphics&list_uids=8607) |

| **ID:GO:0042162      Name: telomeric DNA binding** | | | |
| --- | --- | --- | --- |
| C=30; O=3; E=0.1; R=29.3; PValue=1.4e-04; FDR=1.26e-02 | | | |
| **AC** | **Gene Symbol** | **Gene Name** | **Entrez Gene** |
| P09651 | HNRNPA1 | heterogeneous nuclear ribonucleoprotein A1 | [3178](http://www.ncbi.nlm.nih.gov/sites/entrez?db=gene&cmd=Retrieve&dopt=Graphics&list_uids=3178) |
| P22626 | HNRNPA2B1 | heterogeneous nuclear ribonucleoprotein A2/B1 | [3181](http://www.ncbi.nlm.nih.gov/sites/entrez?db=gene&cmd=Retrieve&dopt=Graphics&list_uids=3181) |
| Q14103 | HNRNPD | heterogeneous nuclear ribonucleoprotein D | [3184](http://www.ncbi.nlm.nih.gov/sites/entrez?db=gene&cmd=Retrieve&dopt=Graphics&list_uids=3184) |

| [**ID:GO:0051082      Name:unfolded protein binding**](http://amigo.geneontology.org/amigo/term/GO:0051082) | | | |
| --- | --- | --- | --- |
| C=105; O=4; E=0.36; R=11.16; PValue=4.35e-04; FDR=2.93e-02 | | | |
| **AC** | **Gene Symbol** | **Gene Name** | **Entrez Gene** |
| P38646 | HSPA9 | heat shock protein family A (Hsp70) member 9 | [3313](http://www.ncbi.nlm.nih.gov/sites/entrez?db=gene&cmd=Retrieve&dopt=Graphics&list_uids=3313) |
| P06748 | NPM1 | nucleophosmin | [4869](http://www.ncbi.nlm.nih.gov/sites/entrez?db=gene&cmd=Retrieve&dopt=Graphics&list_uids=4869) |
| P62937 | PPIA | peptidylprolyl isomerase A | [5478](http://www.ncbi.nlm.nih.gov/sites/entrez?db=gene&cmd=Retrieve&dopt=Graphics&list_uids=5478) |
| P40227 | CCT6A | chaperonin containing TCP1 subunit 6A | [908](http://www.ncbi.nlm.nih.gov/sites/entrez?db=gene&cmd=Retrieve&dopt=Graphics&list_uids=908) |

| **ID:GO:0003727      Name: single-stranded RNA binding** | | | |
| --- | --- | --- | --- |
| C=70; O=3; E=0.24; R=12.56; PValue=1.72e-03; FDR=9.08e-02 | | | |
| **AC** | **Gene Symbol** | **Gene Name** | **Entrez Gene** |
| O60506 | SYNCRIP | synaptotagmin binding cytoplasmic RNA interacting protein | [10492](http://www.ncbi.nlm.nih.gov/sites/entrez?db=gene&cmd=Retrieve&dopt=Graphics&list_uids=10492) |
| P09651 | HNRNPA1 | heterogeneous nuclear ribonucleoprotein A1 | [3178](http://www.ncbi.nlm.nih.gov/sites/entrez?db=gene&cmd=Retrieve&dopt=Graphics&list_uids=3178) |
| P52597 | HNRNPF | heterogeneous nuclear ribonucleoprotein F | [3185](http://www.ncbi.nlm.nih.gov/sites/entrez?db=gene&cmd=Retrieve&dopt=Graphics&list_uids=3185) |

| **ID:GO:0017025      Name: TBP-class protein binding** | | | |
| --- | --- | --- | --- |
| C=21; O=2; E=0.07; R=27.91; PValue=2.29e-03; FDR=9.08e-02 | | | |
| **AC** | **Gene Symbol** | **Gene Name** | **Entrez Gene** |
| P52597 | HNRNPF | heterogeneous nuclear ribonucleoprotein F | [3185](http://www.ncbi.nlm.nih.gov/sites/entrez?db=gene&cmd=Retrieve&dopt=Graphics&list_uids=3185) |
| P62333 | PSMC6 | proteasome 26S subunit, ATPase 6 | [5706](http://www.ncbi.nlm.nih.gov/sites/entrez?db=gene&cmd=Retrieve&dopt=Graphics&list_uids=5706) |

| **ID:GO:0003729      Name: mRNA binding** | | | |
| --- | --- | --- | --- |
| C=170; O=4; E=0.58; R=6.89; PValue=2.61e-03; FDR=9.08e-02 | | | |
| **AC** | **Gene Symbol** | **Gene Name** | **Entrez Gene** |
| Q8NC51 | SERBP1 | SERPINE1 mRNA binding protein 1 | [26135](http://www.ncbi.nlm.nih.gov/sites/entrez?db=gene&cmd=Retrieve&dopt=Graphics&list_uids=26135) |
| P22626 | HNRNPA2B1 | heterogeneous nuclear ribonucleoprotein A2/B1 | [3181](http://www.ncbi.nlm.nih.gov/sites/entrez?db=gene&cmd=Retrieve&dopt=Graphics&list_uids=3181) |
| Q14103 | HNRNPD | heterogeneous nuclear ribonucleoprotein D | [3184](http://www.ncbi.nlm.nih.gov/sites/entrez?db=gene&cmd=Retrieve&dopt=Graphics&list_uids=3184) |
| Q9BWF3 | RBM4 | RNA binding motif protein 4 | [5936](http://www.ncbi.nlm.nih.gov/sites/entrez?db=gene&cmd=Retrieve&dopt=Graphics&list_uids=5936) |

| **ID:GO:0044389      Name: ubiquitin-like protein ligase binding** | | | |
| --- | --- | --- | --- |
| C=285; O=5; E=0.97; R=5.14; PValue=2.7e-03; FDR=9.08e-02 | | | |
| **AC** | **Gene Symbol** | **Gene Name** | **Entrez Gene** |
| P07437 | TUBB | tubulin beta class I | [203068](http://www.ncbi.nlm.nih.gov/sites/entrez?db=gene&cmd=Retrieve&dopt=Graphics&list_uids=203068) |
| P38646 | HSPA9 | heat shock protein family A (Hsp70) member 9 | [3313](http://www.ncbi.nlm.nih.gov/sites/entrez?db=gene&cmd=Retrieve&dopt=Graphics&list_uids=3313) |
| Q9UQ80 | PA2G4 | proliferation-associated 2G4 | [5036](http://www.ncbi.nlm.nih.gov/sites/entrez?db=gene&cmd=Retrieve&dopt=Graphics&list_uids=5036) |
| P60174 | TPI1 | triosephosphate isomerase 1 | [7167](http://www.ncbi.nlm.nih.gov/sites/entrez?db=gene&cmd=Retrieve&dopt=Graphics&list_uids=7167) |
| P55072 | VCP | valosin containing protein | [7415](http://www.ncbi.nlm.nih.gov/sites/entrez?db=gene&cmd=Retrieve&dopt=Graphics&list_uids=7415) |

| **ID:GO:0042287      Name: MHC protein binding** | | | |
| --- | --- | --- | --- |
| C=25; O=2; E=0.09; R=23.44; PValue=3.24e-03; FDR=9.43e-02 | | | |
| **AC** | **Gene Symbol** | **Gene Name** | **Entrez Gene** |
| P07437 | TUBB | tubulin beta class I | [203068](http://www.ncbi.nlm.nih.gov/sites/entrez?db=gene&cmd=Retrieve&dopt=Graphics&list_uids=203068) |
| P55072 | VCP | valosin containing protein | [7415](http://www.ncbi.nlm.nih.gov/sites/entrez?db=gene&cmd=Retrieve&dopt=Graphics&list_uids=7415) |

| **ID:GO:0036002      Name: pre-mRNA binding** | | | |
| --- | --- | --- | --- |
| C=26; O=2; E=0.09; R=22.54; PValue=3.51e-03; FDR=9.43e-02 | | | |
| **AC** | **Gene Symbol** | **Gene Name** | **Entrez Gene** |
| P22626 | HNRNPA2B1 | heterogeneous nuclear ribonucleoprotein A2/B1 | [3181](http://www.ncbi.nlm.nih.gov/sites/entrez?db=gene&cmd=Retrieve&dopt=Graphics&list_uids=3181) |
| Q9BWF3 | RBM4 | RNA binding motif protein 4 | [5936](http://www.ncbi.nlm.nih.gov/sites/entrez?db=gene&cmd=Retrieve&dopt=Graphics&list_uids=5936) |

| ***Cellular Component*** | | | |
| --- | --- | --- | --- |
| **ID:GO:0043209      Name: myelin sheath** | | | |
| C=165; O=7; E=0.62; R=11.31; PValue=2.11e-06; FDR=3.12e-04 | | | |
| **AC** | **Gene Symbol** | **Gene Name** | **Entrez Gene** |
| P31948 | STIP1 | stress induced phosphoprotein 1 | [10963](http://www.ncbi.nlm.nih.gov/sites/entrez?db=gene&cmd=Retrieve&dopt=Graphics&list_uids=10963) |
| P38646 | HSPA9 | heat shock protein family A (Hsp70) member 9 | [3313](http://www.ncbi.nlm.nih.gov/sites/entrez?db=gene&cmd=Retrieve&dopt=Graphics&list_uids=3313) |
| Q99798 | ACO2 | aconitase 2 | [50](http://www.ncbi.nlm.nih.gov/sites/entrez?db=gene&cmd=Retrieve&dopt=Graphics&list_uids=50) |
| Q06830 | PRDX1 | peroxiredoxin 1 | [5052](http://www.ncbi.nlm.nih.gov/sites/entrez?db=gene&cmd=Retrieve&dopt=Graphics&list_uids=5052) |
| P36542 | ATP5C1 | ATP synthase, H+ transporting, mitochondrial F1 complex, gamma polypeptide 1 | [509](http://www.ncbi.nlm.nih.gov/sites/entrez?db=gene&cmd=Retrieve&dopt=Graphics&list_uids=509) |
| P55072 | VCP | valosin containing protein | [7415](http://www.ncbi.nlm.nih.gov/sites/entrez?db=gene&cmd=Retrieve&dopt=Graphics&list_uids=7415) |
| P45880 | VDAC2 | voltage dependent anion channel 2 | [7417](http://www.ncbi.nlm.nih.gov/sites/entrez?db=gene&cmd=Retrieve&dopt=Graphics&list_uids=7417) |

| **ID:GO:0005759      Name: mitochondrial matrix** | | | |
| --- | --- | --- | --- |
| C=423; O=8; E=1.59; R=5.04; PValue=1.33e-04; FDR=7.28e-03 | | | |
| **AC** | **Gene Symbol** | **Gene Name** | **Entrez Gene** |
| P13995 | MTHFD2 | methylenetetrahydrofolate dehydrogenase (NADP+ dependent) 2, methenyltetrahydrofolate cyclohydrolase | [10797](http://www.ncbi.nlm.nih.gov/sites/entrez?db=gene&cmd=Retrieve&dopt=Graphics&list_uids=10797) |
| Q6NVY1 | HIBCH | 3-hydroxyisobutyryl-CoA hydrolase | [26275](http://www.ncbi.nlm.nih.gov/sites/entrez?db=gene&cmd=Retrieve&dopt=Graphics&list_uids=26275) |
| P38646 | HSPA9 | heat shock protein family A (Hsp70) member 9 | [3313](http://www.ncbi.nlm.nih.gov/sites/entrez?db=gene&cmd=Retrieve&dopt=Graphics&list_uids=3313) |
| Q99798 | ACO2 | aconitase 2 | [50](http://www.ncbi.nlm.nih.gov/sites/entrez?db=gene&cmd=Retrieve&dopt=Graphics&list_uids=50) |
| P55809 | OXCT1 | 3-oxoacid CoA-transferase 1 | [5019](http://www.ncbi.nlm.nih.gov/sites/entrez?db=gene&cmd=Retrieve&dopt=Graphics&list_uids=5019) |
| P36542 | ATP5C1 | ATP synthase, H+ transporting, mitochondrial F1 complex, gamma polypeptide 1 | [509](http://www.ncbi.nlm.nih.gov/sites/entrez?db=gene&cmd=Retrieve&dopt=Graphics&list_uids=509) |
| Q04837 | SSBP1 | single stranded DNA binding protein 1 | [6742](http://www.ncbi.nlm.nih.gov/sites/entrez?db=gene&cmd=Retrieve&dopt=Graphics&list_uids=6742) |
| P45880 | VDAC2 | voltage dependent anion channel 2 | [7417](http://www.ncbi.nlm.nih.gov/sites/entrez?db=gene&cmd=Retrieve&dopt=Graphics&list_uids=7417) |

| [**ID:GO:0005913      Name:cell-cell adherens junction**](http://amigo.geneontology.org/amigo/term/GO:0005913) | | | |
| --- | --- | --- | --- |
| C=318; O=7; E=1.19; R=5.87; PValue=1.47e-04; FDR=7.28e-03 | | | |
| **AC** | **Gene Symbol** | **Gene Name** | **Entrez Gene** |
| P29692 | EEF1D | eukaryotic translation elongation factor 1 delta | [1936](http://www.ncbi.nlm.nih.gov/sites/entrez?db=gene&cmd=Retrieve&dopt=Graphics&list_uids=1936) |
| P13639 | EEF2 | eukaryotic translation elongation factor 2 | [1938](http://www.ncbi.nlm.nih.gov/sites/entrez?db=gene&cmd=Retrieve&dopt=Graphics&list_uids=1938) |
| P04075 | ALDOA | aldolase, fructose-bisphosphate A | [226](http://www.ncbi.nlm.nih.gov/sites/entrez?db=gene&cmd=Retrieve&dopt=Graphics&list_uids=226) |
| Q8NC51 | SERBP1 | SERPINE1 mRNA binding protein 1 | [26135](http://www.ncbi.nlm.nih.gov/sites/entrez?db=gene&cmd=Retrieve&dopt=Graphics&list_uids=26135) |
| Q06830 | PRDX1 | peroxiredoxin 1 | [5052](http://www.ncbi.nlm.nih.gov/sites/entrez?db=gene&cmd=Retrieve&dopt=Graphics&list_uids=5052) |
| P52907 | CAPZA1 | capping actin protein of muscle Z-line alpha subunit 1 | [829](http://www.ncbi.nlm.nih.gov/sites/entrez?db=gene&cmd=Retrieve&dopt=Graphics&list_uids=829) |
| Q9Y265 | RUVBL1 | RuvB like AAA ATPase 1 | [8607](http://www.ncbi.nlm.nih.gov/sites/entrez?db=gene&cmd=Retrieve&dopt=Graphics&list_uids=8607) |

| **ID:GO:0009295      Name: nucleoid** | | | |
| --- | --- | --- | --- |
| C=45; O=3; E=0.17; R=17.78; PValue=6.16e-04; FDR=2.28e-02 | | | |
| **AC** | **Gene Symbol** | **Gene Name** | **Entrez Gene** |
| P38646 | HSPA9 | heat shock protein family A (Hsp70) member 9 | [3313](http://www.ncbi.nlm.nih.gov/sites/entrez?db=gene&cmd=Retrieve&dopt=Graphics&list_uids=3313) |
| Q04837 | SSBP1 | single stranded DNA binding protein 1 | [6742](http://www.ncbi.nlm.nih.gov/sites/entrez?db=gene&cmd=Retrieve&dopt=Graphics&list_uids=6742) |
| P45880 | VDAC2 | voltage dependent anion channel 2 | [7417](http://www.ncbi.nlm.nih.gov/sites/entrez?db=gene&cmd=Retrieve&dopt=Graphics&list_uids=7417) |

| [**ID:GO:0005681      Name:spliceosomal complex**](http://amigo.geneontology.org/amigo/term/GO:0005681) | | | |
| --- | --- | --- | --- |
| C=173; O=4; E=0.65; R=6.16; PValue=3.81e-03; FDR=1.13e-01 | | | |
| **AC** | **Gene Symbol** | **Gene Name** | **Entrez Gene** |
| O60506 | SYNCRIP | synaptotagmin binding cytoplasmic RNA interacting protein | [10492](http://www.ncbi.nlm.nih.gov/sites/entrez?db=gene&cmd=Retrieve&dopt=Graphics&list_uids=10492) |
| P09651 | HNRNPA1 | heterogeneous nuclear ribonucleoprotein A1 | [3178](http://www.ncbi.nlm.nih.gov/sites/entrez?db=gene&cmd=Retrieve&dopt=Graphics&list_uids=3178) |
| P22626 | HNRNPA2B1 | heterogeneous nuclear ribonucleoprotein A2/B1 | [3181](http://www.ncbi.nlm.nih.gov/sites/entrez?db=gene&cmd=Retrieve&dopt=Graphics&list_uids=3181) |
| P52597 | HNRNPF | heterogeneous nuclear ribonucleoprotein F | [3185](http://www.ncbi.nlm.nih.gov/sites/entrez?db=gene&cmd=Retrieve&dopt=Graphics&list_uids=3185) |

| ***Pathway (KEGG database)*** | | | |
| --- | --- | --- | --- |
| **ID:hsa01200      Name: Carbon metabolism - Homo sapiens (human)** | | | |
| C=114; O=5; E=0.49; R=10.15; PValue=1.13e-04; FDR=3.43e-02 | | | |
| **AC** | **Gene Symbol** | **Gene Name** | **Entrez Gene** |
| P04075 | ALDOA | aldolase, fructose-bisphosphate A | [226](http://www.ncbi.nlm.nih.gov/sites/entrez?db=gene&cmd=Retrieve&dopt=Graphics&list_uids=226) |
| P04406 | GAPDH | glyceraldehyde-3-phosphate dehydrogenase | [2597](http://www.ncbi.nlm.nih.gov/sites/entrez?db=gene&cmd=Retrieve&dopt=Graphics&list_uids=2597) |
| Q6NVY1 | HIBCH | 3-hydroxyisobutyryl-CoA hydrolase | [26275](http://www.ncbi.nlm.nih.gov/sites/entrez?db=gene&cmd=Retrieve&dopt=Graphics&list_uids=26275) |
| Q99798 | ACO2 | aconitase 2 | [50](http://www.ncbi.nlm.nih.gov/sites/entrez?db=gene&cmd=Retrieve&dopt=Graphics&list_uids=50) |
| P60174 | TPI1 | triosephosphate isomerase 1 | [7167](http://www.ncbi.nlm.nih.gov/sites/entrez?db=gene&cmd=Retrieve&dopt=Graphics&list_uids=7167) |

| **ID:hsa01230      Name: Biosynthesis of amino acids - Homo sapiens (human)** | | | |
| --- | --- | --- | --- |
| C=75; O=4; E=0.32; R=12.34; PValue=2.8e-04; FDR=4.24e-02 | | | |
| **AC** | **Gene Symbol** | **Gene Name** | **Entrez Gene** |
| P04075 | ALDOA | aldolase, fructose-bisphosphate A | [226](http://www.ncbi.nlm.nih.gov/sites/entrez?db=gene&cmd=Retrieve&dopt=Graphics&list_uids=226) |
| P04406 | GAPDH | glyceraldehyde-3-phosphate dehydrogenase | [2597](http://www.ncbi.nlm.nih.gov/sites/entrez?db=gene&cmd=Retrieve&dopt=Graphics&list_uids=2597) |
| Q99798 | ACO2 | aconitase 2 | [50](http://www.ncbi.nlm.nih.gov/sites/entrez?db=gene&cmd=Retrieve&dopt=Graphics&list_uids=50) |
| P60174 | TPI1 | triosephosphate isomerase 1 | [7167](http://www.ncbi.nlm.nih.gov/sites/entrez?db=gene&cmd=Retrieve&dopt=Graphics&list_uids=7167) |

| ***Pathway (Panther database)*** | | | |
| --- | --- | --- | --- |
| **ID:P00024      Name: Glycolysis** | | | |
| C=17; O=3; E=0.08; R=36.53; PValue=5.5e-05; FDR=6.22e-03 | | | |
| **AC** | **Gene Symbol** | **Gene Name** | **Entrez Gene** |
| P04075 | ALDOA | aldolase, fructose-bisphosphate A | [226](http://www.ncbi.nlm.nih.gov/sites/entrez?db=gene&cmd=Retrieve&dopt=Graphics&list_uids=226) |
| P04406 | GAPDH | glyceraldehyde-3-phosphate dehydrogenase | [2597](http://www.ncbi.nlm.nih.gov/sites/entrez?db=gene&cmd=Retrieve&dopt=Graphics&list_uids=2597) |
| P60174 | TPI1 | triosephosphate isomerase 1 | 7167 |

AC= Accession number in Swiss-Prot/UniProtKB (http://www.uniprot.org/).

C=The number of reference genes in the category

O=The number of genes in the user gene list and also in the category

E=The expected number in the category

R= Ratio of enrichment

PValue=*p*-value from hyergeometric test

FDR=false discovery rate from Benjamini and Hochberg (BH) test
